# Economically viable co-production of methanol and sulfuric acid via direct methane oxidation

**DOI:** 10.1038/s42004-023-01080-4

**Published:** 2023-12-20

**Authors:** Jaehyung Im, Seok-Hyeon Cheong, Huyen Tran Dang, Nak-Kyoon Kim, Sungwon Hwang, Ki Bong Lee, Kyeongsu Kim, Hyunjoo Lee, Ung Lee

**Affiliations:** 1 Clean Energy Research Center, Korea Institute of Science and Technology (KIST), 02792 Seoul, Republic of Korea; 2 Department of Chemical and Biological Engineering, Korea University, Seoul, 02841 Republic of Korea; 3 Division of Energy & Environmental Technology, KIST School, University of Science and Technology, 02792 Seoul, Republic of Korea; 4 Advanced Analysis Center, Korea Institute of Science and Technology, Seoul, 02792 Republic of Korea; 5 Department of Chemical Engineering, Inha University, Incheon, Republic of Korea

**Keywords:** Energy infrastructure, Catalysis, Natural gas, Chemical engineering, Process chemistry

## Abstract

The direct oxidation of methane to methanol has been spotlighted research for decades, but has never been commercialized. This study introduces cost-effective process for co-producing methanol and sulfuric acid through a direct oxidation of methane. In the initial phase, methane oxidation forms methyl bisulfate (CH_3_OSO_3_H), then transformed into methyl trifluoroacetate (CF_3_CO_2_CH_3_) via esterification, and hydrolyzed into methanol. This approach eliminates the need for energy-intensive separation of methyl bisulfate from sulfuric acid by replacing the former with methyl trifluoroacetate. Through the superstructure optimization, our sequential process reduces the levelized cost of methanol to nearly two-fold reduction from the current market price. Importantly, this process demonstrates adaptability to smaller gas fields, assuring its economical operation across a broad range of gas fields. The broader application of this process could substantially mitigate global warming by utilizing methane, leading to a significantly more sustainable and economically beneficial methanol industry.

## Abstract (web summary)

Conversion of methane to methanol via methanol derivatives such as methyl bisulfate (MBS) allows to achieve high selectivity and yield, but separating MBS from oxidizing agents such as sulfuric acid is an energy-intense step. Here, the authors eliminate the need for separation of MBS from sulfuric acid by replacing the former with methyl trifluoroacetate, which is subsequently hydrolyzed into high-purity methanol.

## Introduction

Methane is the most abundant energy source and an important hydrocarbon feedstock for producing fuels and chemicals. It is also considered a transitional fuel that alleviates the current reliance on finite crude oil reserves for energy and chemical synthesis^1^. Recent technical advances in shale gas exploitation and drilling have significantly increased methane production^2^. Consequently, the utilization of methane for fuel and chemical production has received considerable attention.

Until now, the only available route for commercial methane utilization producing value-added liquid products was an energy-intensive indirect conversion that includes syngas production followed by a series of refinement processes^3^. Although the indirect conversion of methane is mature enough to be widely applied in chemical production^4^, such technologies are not adequate for local and small-scale facilities, such as remote oil fields. Consequently, 143 billion m3 of natural gas has been flared for over the past fifteen years, wasting potential feedstock and causing greenhouse gas emissions^5,6^.

To overcome the drawbacks of indirect conversion of methane, many studies have focused on the direct conversion of methane-producing methanol. Consequently, numerous systems such as gas-phase reactions using Cu-zeolite catalysts^7–16^, using plasma without catalysts^17,18^, in-situ generation of H_2_O_2_ using H_2_ and O_2_^19–24^, and diverse catalytic systems^25–30^ that can selectively oxidize methane to methanol have been proposed recent decades (see Supplementary Note 1 and Supplementary Table 2). However, none of the suggested reaction systems have been commercialized because of their low methanol selectivity and yields^1^. This is because the direct conversion reaction requires high energy to break the covalent C-H bond (438.8 kJ/mol) in methane. Accordingly, highly reactive reagents and harsh reaction conditions are applied to activate the C-H bond, which in turn leads to further oxidation of the reaction product, methanol, to carbon monoxide and dioxide. To address this problem, several studies have focused on the synthesis of methanol derivatives such as methyl bisulfate^31–35^, methyl bromide^36^, and methyl trifluoroacetate^37–40^, which are more stable than methane in the oxidation conditions^1^.

In the context of converting methane into methanol derivatives, the use of methyl bisulfate (MBS, CH_3_OSO_3_H) as an intermediate offers several advantages. Firstly, its synthesis through the oxidation of methane with SO_3_ in H_2_SO_4_ media is a cost-effective method. Additionally, MBS exhibits a high product yield, making it an attractive option for further processing into methanol^1^. Periana et al. reported the synthesis of MBS using a bipyrimidyl-bonded platinum catalyst, (bpym)PtCl_2_, which showed a methane conversion of 72% and MBS selectivity of 81%^33^. Although the activity of the Periana catalyst was surprising compared to those of other catalyst systems reported at that time, its performance was still insufficient for industrial applications; the maximum turnover number (TON) of the catalyst was 500. Recently, Pt-black, K_2_PtCl_4_, and (DMSO)_2_PtCl_2_ catalyst systems have shed light on the industrial potential of methane activation to MBS by attaining high reaction performance under relatively mild conditions^31,35,41,42^. T. Zimmermann et al. reported K_2_PtCl_4_ could convert methane to MBS with a TOF over 25,000/h, which is enough for commercial process^35^. Dang et al. developed (DMSO)_2_PtCl_2_ catalyst having >94% selectivity with >84% MBS yield^41^, which was followed by deactivation-free Pt-black catalyst with similar activity^42^. Nevertheless, the practical application of these catalysts to industrial methanol production was still challenging because of the separation of MBS from the sulfuric acid solution^35^.

The separation of MBS from sulfuric acid requires distillation at high temperatures or depressurization to 100 mbar, which in turn decomposes MBS into SO_3_, dimethyl ether, and dimethylsulfate^42^. Michalkiewicz et al. suggested the use of membranes for the separation of products^43^, however, the functionality of the membrane under such strong acidic conditions is still questionable. Furthermore, addition of water to the mixture of MBS and sulfuric acid to hydrolyze MBS to methanol wastes large amount of diluted sulfuric acid. According to Ahlquist et al., methanol concentration cannot be higher than 10 µM in sulfuric acid as methanol undergoes additional oxidation^44^. Accordingly, the produced MBS should be separated from sulfuric acid before it is converted into methanol^45–47^.

The direct methanol synthesis method presented here is fundamentally different from the above-mentioned approaches. The proposed reaction pathway directly converts methane to methanol through oxidation, esterification, and hydrolysis. The benefits of this reaction sequence are significant, as the second step of esterification can alleviate the burden of separating methyl bisulfate (MBS) from the sulfuric acid solution. This is due to the relatively lower boiling point of methyl trifluoroacetate (Me-TFA), 43.5 °C, compared to that of MBS, which exceeds 170 °C. Subsequently, Me-TFA is hydrolyzed to methanol and trifluoroacetic acid (TFA), with the latter being recycled for the synthesis of Me-TFA. The economic feasibility and carbon footprint of the proposed sequential reaction was evaluated through process design and optimization. Numerous process alternatives inherently included in the proposed reaction are evaluated using a superstructure and machine learning-based optimization method. The results of this study reveal that the methanol price can be reduced to $203 ton^−1^, which is approximately twice lower than the current price, when the sulfuric acid price is maintained at its market price. Additionally, the proposed process can be applied to small gas fields, which can be used to produce 16,000-ton methanol per year while meeting economic viability.

## Results

### Experimental investigation of sequential reaction

**Fig. 1:**
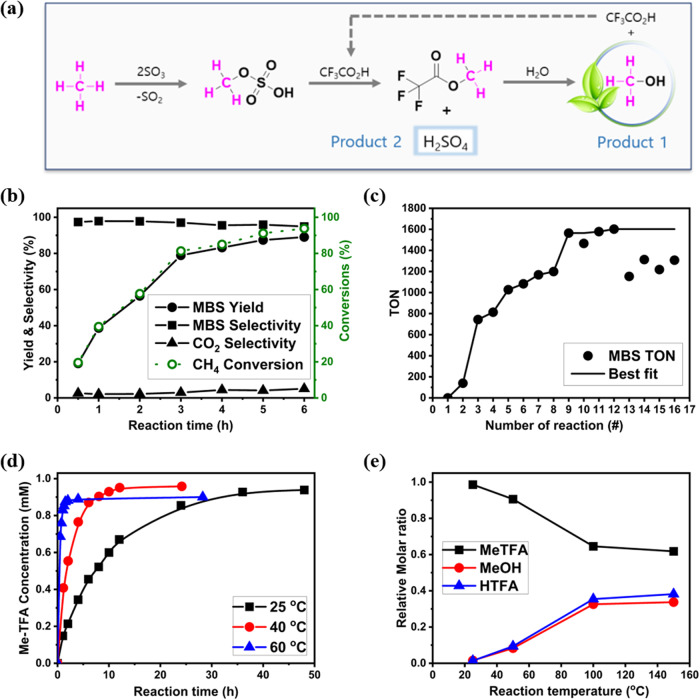
Experimental results of direct methanol synthesis method. **a** Reaction scheme for the proposed direct methanol synthesis method. **b** Effect of reaction time on the Pt-black-catalyzed methane oxidation to methyl bisulfate (MBS). Conditions: 3 mg of Pt-catalyst (0.015 mmol), 30 g of oleum (20 wt% SO_3_), 25 bar of CH_4_, 180 °C. **c** Bayesian optimization. Conditions: 5 mg of Pt-catalyst (0.025 mmol), 35 bar of CH_4_, 3 h **d** Time dependence of the reaction between methyl bisulfate (MBS) and trifluoroacetic acid (TFA) at different temperatures on methyl trifluoroacetate (Me-TFA) formation equilibrium state. Conditions: 0.5 g of liquid product (0.54 mmol of MBS in H_2_SO_4_), 0.3 g (2.24 mmol) of CF_3_CO_2_D **e** Temperature scanning experiments on methyl trifluoroacetate (Me-TFA) hydrolysis reaction.

The sequential reaction starts with methane oxidation with SO_3_ to synthesize MBS in the sulfuric acid media, which is followed by transferring the methyl group of MBS to Me-TFA (Fig. 1a). It is worth noting that previous research has investigated direct Me-TFA synthesis from methane by conducting oxidation in TFA media^37–40,48,49^. However, due to low methane conversion yields and significant solvent decomposition during oxidation, further catalyst development is required to advance this method beyond academic interest^40^. The net reaction of Fig. 1a can be expressed by Eq. (1), whereby one mole of methane reacts with two moles of SO_3_ and one mole of water to produce methanol and sulfuric acid as follows. It is noteworthy that the use of SO_3_ oxidant enables the co-production of both methanol and sulfuric acid as valuable economic products.

1 $${{{{{{\rm{CH}}}}}}}_{4}+2{{{{{{\rm{SO}}}}}}}_{3}+{{{{{{\rm{H}}}}}}}_{2}O\to {{{{{{\rm{CH}}}}}}}_{3}{{{{{\rm{OH}}}}}}+{{{{{{\rm{H}}}}}}}_{2}{{{{{{\rm{SO}}}}}}}_{4}+{{{{{{\rm{SO}}}}}}}_{2}$$

The methane oxidation is the most crucial step because the yields of subsequent esterification, and hydrolysis reaction are mainly depended on the methane conversion to MBS. The reaction equations for the methane oxidation to MBS and the side product CO_2_ formation are shown in Eqs. (2) and (3), respectively^41^.

2 $${{{{{{\rm{CH}}}}}}}_{4}+{2{{{{{\rm{SO}}}}}}}_{3}\to {{{{{{\rm{CH}}}}}}}_{3}{{{{{{\rm{OSO}}}}}}}_{3}{{{{{\rm{H}}}}}}+{{{{{{\rm{SO}}}}}}}_{2}$$

3 $${{{{{{\rm{CH}}}}}}}_{4}+{6{{{{{\rm{SO}}}}}}}_{3}\to {{{{{{\rm{CO}}}}}}}_{2}+{4{{{{{\rm{SO}}}}}}}_{2}+{2{{{{{{\rm{H}}}}}}}_{2}{{{{{\rm{SO}}}}}}}_{4}$$

To maximize the MBS yield, we carried out methane oxidation reaction experiment using Gaussian process Bayesian optimization (GPBO)^50,51^. GPBO captures the correlation between the input (i.e., experimental conditions) and output (i.e., reaction yield), and builds a surrogate model based on given data. Then, the GPBO suggests the next experimental condition decided by optimizing the surrogate model, and in turn, the acquired experimental data is used for updating the surrogate model. This way, the GPBO extensively focuses on the region of interests and the optimum operating condition can be economically obtained.

Methane oxidation was conducted between 180 and 235 °C for 3 h as described in the “Methods” section. Among the various Pt-catalyst reported, Pt-black was chosen due to its stability and reusability^42^. Figure 1b shows the impacts of the reaction time on the methane oxidation efficiency at 180 °C and 20 wt% of oleum concentration. The catalytic activity of Pt-black gradually increased over reaction time. At 30 min of reaction, 19.6% of CH_4_ was converted into the oxidation products (MBS and CO_2_) and the yield of MBS was 19.1%. As the reaction time increases up to 3 h, the conversion of CH_4_ reached 81.3%, which in turn increased to 93.8% after 6 h of reaction. The formation of CO_2_ was gradually increased during the reaction; at 3 h, the CO_2_ selectivity was 2.9%, which was increased to 5.1% by the reaction of 6 h. Supplementary Fig. 2 shows the impacts of the catalyst concentration and the reaction time on the methane oxidation efficiency. As shown in Supplementary Fig. 2, when the catalyst concentration is low (0.31 mM), 26.4% of MBS was formed and gradually increased up to 81.3% at the catalyst concentration of 0.94 mM. The use of catalyst above 0.94 mM did not significantly affect the oxidation results. This is because Pt-black dissolution in oleum (20 wt%) gets saturated.

The optimum reaction temperature and oleum concentration obtained through GPBO is presented in Fig. 1c and Supplementary Table 1. The nonconvex response of TON on experimental conditions was found within the search domain (see Supplementary Fig. 3a). However, a Gaussian nonlinear regression model was successfully applied to optimize the operating conditions. The predicted optimum reaction temperature and oleum concentration are 200 °C and 33 wt%, respectively. It is noteworthy that the TON of the oxidation reaction exhibits varying responses depending on the reaction conditions based on the optimum point. In Supplementary Fig. 3b, which illustrates the regression of TON as a function of the reaction temperature and oleum concentration, the TON appears to be highly correlated with the reaction temperature when the temperature is lower than the optimal condition. Conversely, when the temperature exceeds the optimal condition, both reaction conditions have an impact on TON, and, in particular, the reaction temperature is negatively correlated with TON.

As shown in Fig. 1d, the experiments to calculate equilibrium constants of the esterification reaction (Eq. (4)) have been carried out at 25, 40, and 60 °C and the equilibrium constants were calculated as 6.71, 6.43, and 6.11 for the esterification reactions. The tendency of the equilibrium constants indicates that the esterification of MBS and TFA is an exothermic reaction.

4 $${{{{{{\rm{CH}}}}}}}_{3}{{{{{{\rm{OSO}}}}}}}_{3}{{{{{\rm{H}}}}}}+{{{{{{\rm{CF}}}}}}}_{3}{{{{{\rm{COOH}}}}}}\to {{{{{{\rm{CF}}}}}}}_{3}{{{{{{\rm{CO}}}}}}}_{2}{{{{{{\rm{CH}}}}}}}_{3}+{{{{{{{\rm{H}}}}}}}_{2}{{{{{\rm{SO}}}}}}}_{4}$$

5 $${{{{{{\rm{H}}}}}}}_{2}{{{{{{\rm{SO}}}}}}}_{4}\to {{{{{{\rm{SO}}}}}}}_{3}+{{{{{{\rm{H}}}}}}}_{2}{{{{{\rm{O}}}}}}$$

The esterification process undertaken through batch reaction yielded a maximum MBS conversion of approximately 73% (see Supplementary Table 3). The optimal conversion was attained at a temperature of 40 °C. This outcome can be attributed to the fact that the equilibrium concentration of Me-TFA decreases with an increase in temperature. While it may be possible to achieve higher MBS conversion with lower temperature, this would come at a cost to the economic viability of the process, as it would require a larger reactor volume due to the slow reaction kinetics. In fact, a lower MBS conversion rate was observed even with twice the time (48 h) at a lower temperature of 25 °C. However, the esterification reaction requires less than 2 h to converge to its equilibrium state when the experiment is conducted over 60 °C. Considering these results, the esterification reaction was designed to be operated over 60 °C. The conversion efficiency of MBS can be further enhanced by employing reactive distillation column techniques (see Supplementary Note 2). Notably, in a reactive distillation setup, the removal of Me-TFA from the feed stream was observed to result in an 86% conversion of MBS (see Supplementary Table 4).

From the regression analysis, the correlation between the equilibrium constant, *K*_*eq,est*_, and the reaction temperature, *T*, was obtained as Eq. (6).

6 $${{{{{\mathrm{ln}}}}}}({K}_{{eq},{est}})=8.339-1.398{{{{\mathrm{ln}}}}}(T)$$

The equation for *K*_*eq,est*_ was adopted to design the reactive distillation column producing Me-TFA, and the accompanying decomposition of sulfuric acid (Eq. (5)) was assumed to be in Gibbs equilibrium.

In order to determine the correlation for the hydrolysis reaction between the equilibrium constant, *K*_*eq*,*hyd*,_ and the reaction temperature, the hydrolysis products were measured between 20 °C and 150 °C (Eq. (7)). The experimental results are presented in Fig. 1e. The hydrolysis reaction seems strongly affected by the reaction temperatures. At 25 °C, the efficiency of this hydrolysis reaction was quite low producing a negligible amount of methanol (1.5%) and TFA (1.3%). As the temperature increases up to 100 °C, the proportions of methanol and TFA gradually grow up to 32% and 35%. Further increase in the reaction temperature over 100 °C does not show significant impact on the reaction performances. According to the hydrolysis reaction equation, the obtained amount of methanol and TFA should be equivalent to each other, however, as can be seen in Fig. 1e, from the temperature range over 100 °C, deviation in the amount of products can be observed. This might be caused by dehydration of methanol to dimethyl ether in acidic solution can occur at high reaction temperatures^52^. Although we did not isolate and analyze dimethyl ether, it is assumed that dimethyl ether was formed as much as the deficient amount of methanol in Fig. 1e in order to confirm the mass balance of the experiment. According to the experimental data, we regress the equilibrium constant in terms of temperature as Eq. (8), where *T* is the reaction temperature.

7 $${{{{{{\rm{CF}}}}}}}_{3}{{{{{{\rm{CO}}}}}}}_{2}{{{{{{\rm{CH}}}}}}}_{3}+{{{{{{\rm{H}}}}}}}_{2}{{{{{\rm{O}}}}}}\to {{{{{{\rm{CF}}}}}}}_{3}{{{{{{\rm{CO}}}}}}}_{2}{{{{{\rm{H}}}}}}+{{{{{{\rm{CH}}}}}}}_{3}{{{{{\rm{OH}}}}}}$$

8 $${{{{{\mathrm{ln}}}}}}\left({K}_{{eq},{hyd}}\right)=-189.05+31.903{{{{\mathrm{ln}}}}}(T)$$

### Methanol co-production process design

The proposed direct conversion reaction yielded promising results for the direct conversion of methane to methanol. However, to evaluate the economic viability, a process-level assessment is necessary, taking into account factors such as product separation, raw material recycling, and auxiliary operations. To do this, we used a superstructure-based process design and optimization methodology, as shown in Supplementary Fig. 8a. Superstructures encompass various process design options, and using proper optimization can help achieve an optimal design. This method also allows a detailed analysis of the proposed reaction system, by quantifying uncertainties originated from different process alternatives.

Our superstructure consists of 730 potential process configurations, involving 10 binary integer variables that determine the process configuration, and 9 continuous variables that set the optimal operating conditions. Despite the ability of superstructure optimization to concurrently identify the optimal process configuration and operating conditions simultaneously, this approach is computationally challenging for process design due to its large search space and nonconvex domain. To address this, we implemented a hybrid method of variable decomposition method integrated with a Gaussian process Bayesian optimization, which is a machine learning-based optimization solver as shown in Supplementary Fig. 8b. All the calculations for the optimization procedure have been automated by the Aspen Plus – MATLAB interface which allows MATLAB to access the simulation data of Aspen Plus. Based on the flowsheet model simulation using Aspen Plus, an economic analysis was carried out using MATLAB.

The proposed optimization method first determines the optimal process configuration using a hybrid method combining genetic algorithm^53^ and Bayesian optimization. Although the process design attained is yet to be optimal and requires additional optimization to fine-tune continuous variables, the configuration is fixed as the optimal one in this step. The process operating variables were then divided into subgroups based on the binary interaction between the two variables. The binary interactions were calculated using a two-level factorial design, and the variables were then classified into two clusters using a hierarchical clustering method with a dendrogram^54^. As the variables contained in each cluster are gathered based on the proximity of their impact on the objective function value, the variables in different clusters can be considered irrelevant to each other. Accordingly, each variable cluster is sequentially optimized to consider a smaller number of optimization variables at a time and obtain the optimum process design within an affordable computation time. In addition, the Gaussian process Bayesian optimization method was adopted to efficiently attain the optimal solution^51,55,56^.

**Fig. 2:**
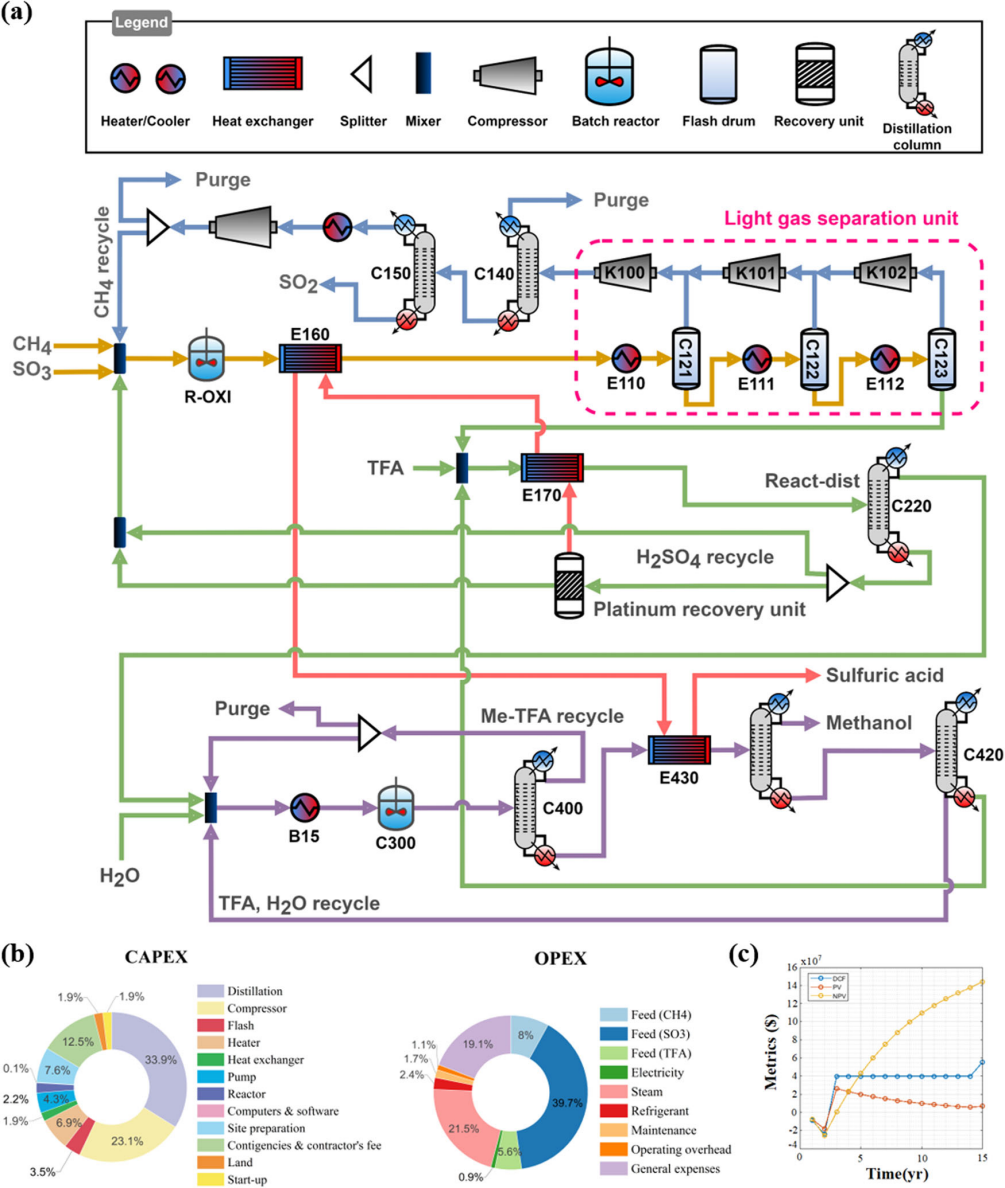
Optimization result and economic analysis of methanol synthesis process. **a** Optimal process configuration resulted from superstructure optimization. The dotted squared area indicates the 3 stage consecutive flash units to recycle methane. **b** Capital expenditure (CAPEX) and operational expenditure (OPEX) breakdown of optimum process design. **c** Discounted cash flow (DCF), present value (PV) and net present value (NPV) over 15 years for optimum process design.

The optimal process configuration identified through superstructure optimization is shown in Fig. 2a. In the oxidation section, which is indicated by yellow lines, methane and SO_3_ reacted to produce MBS and CO_2_. A constant temperature gas induction stirred-tank reactor was utilized for the oxidation reaction, and the conversion and selectivity were directly obtained from lab scale experiments (see Supplementary Fig. 2). The optimum process recovers unreacted raw materials using a three-stage flash column rather than a distillation column and recycles the unreacted raw materials back to the oxidation reactor through a CO_2_ removal unit. The by-product of the oxidation reaction (i.e. SO_2_) is recycled back to the oxidation reactor through the SO_2_ oxidation unit to reduce the raw metric cost. The produced MBS is then introduced to a reactive distillation column to produce Me-TFA and sulfuric acid, where the esterification reaction and sulfuric acid separation take place at the same time. Me-TFA is the light substance in the product mixture from esterification; thus, it can be easily separated from sulfuric acid, thereby avoiding the MBS purification issues raised in previous studies^42–44^. The dissolved Pt in the bottom sulfuric acid stream was concentrated in a platinum recovery unit and recycled to the oxidation reactor. Finally, the Me-TFA hydrolysis is converted to methanol in a hydrolysis reactor.

In the hydrolysis reaction, which is indicated by purple lines, Me-TFA was converted to methanol and TFA with the aid of water in a hydrolysis reactor. The recovered TFA was then recycled back to the esterification reaction after water separation to close the TFA loop. It is worth mentioning that both the continuous stirred-tank reactor and the reactive distillation system were considered options for esterification and hydrolysis reactions. As the reaction performances of esterification and hydrolysis are more sensitive to chemical concentrations and temperature than oxidation, the adoption of reactive species would be helpful in increasing the efficiency of esterification and hydrolysis.

In the optimum process, the conversion of the overall system was maximized using a reactive distillation column for the esterification reaction. This is because the liquid Me-TFA product is continuously removed from the reactant in the reactive distillation column, and thus, the equilibrium of the esterification reaction moves forward to produce more Me-TFA. Interestingly, the optimum strategy for the hydrolysis reaction is not reactive distillation, but a sequential CSTR reactor and separation unit. In the esterification reaction, as the boiling point of the product, Me-TFA, is significantly lower than that of other chemicals, the product can be selectively separated in the distillation column. In contrast, in the hydrolysis reaction, separation of the feed, Me-TFA, and the product, methanol, requires intensive energy input owing to their close boiling points. Thus, the adoption of a reactive distillation column has little impact on moving the hydrolysis equilibrium forward; rather, it increases the size required for methanol separation, resulting in an increase in cost. To recover the unreacted methane, the superstructure optimization selected flash separation instead of a distillation column as methane separation does not require a thermal separation unit, which consumes a substantially larger amount of energy than flash units^7^.

The results of the economic analysis show that OPEX is dominant over CAPEX. Over 90% of the production cost originates from the process operation because of expensive raw materials and extensive steam consumption. As shown in Fig. 2b, the SO_3_ feed costs account for approximately 40% of the OPEX. The excessive cost of SO_3_ feed is primarily because we assume that it is supplied from the commercial market. Thus, the economic feasibility of the proposed system would be further improved if SO_3_ could be supplied from a cheap source, such as power plant waste. Figure 2c shows the cash flow diagram of the optimum process design. As indicated in the figure, the proposed design can achieve a positive NPV of $144 million, and its pay-out time is calculated as a year. The proposed process is particularly competitive for methanol production because it co-produces sulfuric acid, compensating for 93% of the operating cost. The levelized cost of methanol can be as low as $203 ton^−1^, which is relatively low considering the current methanol market price ($270–$450).

### Analysis of suboptimal process configurations

In addition to the analysis of the optimal structure, the analysis was also performed on all alternative cases obtained during the optimization to ensure the reliability of the optimal structure. Supplementary Fig. 6 illustrates the *t*-distributed stochastic neighbor embedding (*t*-SNE) results for the collected data obtained during optimization. *t*-SNE is a nonlinear dimension-reducing algorithm capable of visualizing high dimensional vectors^57^. The large distance between the centers of the two clusters indicates that the NPV calculation results show large differences. Supplementary Fig. 6a shows that all optimization data can be categorized into seven different clusters. As the integer variables have decided the process configuration which has the most dominant impact on economic feasibility, each cluster contains a distinguishable integer variable set except for clusters #5 and #7. As shown in Supplementary Fig. 6c, which presents the simplified process configurations corresponding to each cluster, clusters #5 and #7 use a distillation column to recycle unreacted methane. In this case, the economic feasibility can be largely changed by the amount of energy input to separate methane; therefore, clusters #5 and #7 are dissected, even though they share the same configuration. Each group has a different combination of three configuration variable sets, which consist of the use of a distillation column for separation of CO_2_ and methane, a type of unit operation used in esterification, and separation column sequences for the separation of Me-TFA and methanol.

Among the available process alternatives, product separation column sequencing to purify methanol yielded the longest distance in *t*-SNE. When both methanol and Me-TFA were recovered as a light product in the first column (Supplementary Fig. 6c, #7), the total steam consumption increased by 15–20% compared with the #2 cluster, and even the purity of the methanol product deteriorated, resulting in the loss of expensive TFA. As TFA cannot be neatly separated as a light product under feasible operating conditions, #7 alternative column sequencing limits methanol purity in the next column. Alternative #7 imposes an excessive energetic burden on the first column, and thereby its NPV is significantly lower than that #2 cluster in which column sequencing is supposed to separate Me-TFA first.

The use of reactive distillation (Supplementary Fig. 6c, #6) and CSTR (Supplementary Fig. 6c, #7) is another feature that can clearly distinguish the corresponding cluster through *t*-SNE. As mentioned earlier, the conversion of the esterification reaction is limited to approximately 28% in the CSTR, but that of reactive distillation can be 99% due to the simultaneous occurrence of the reaction and product separation. In the consecutive CSTR and distillation processes, the unreacted feed, mainly MBS and TFA, inevitably flows to the next unit operations, resulting in an increase in the overall process flow rate and heat duty.

As shown in the rightmost column of Supplementary Fig. 6c, cluster #6 contains two different alternatives that can be identified depending on whether a distillation column can be used for methane recycling and CO_2_ removal. The recycling stream from the oxidation reactor contains about 10.7% methane, so purging this stream leads to a large amount of economic loss. Therefore, it is preferable to include a separation unit in the optimal process in terms of economic feasibility. However, the recycle stream contains a small amount of CO_2_; thus, a similar and small energy input is required to separate it, regardless of the use of a distillation column or flash unit.

Among the clusters in Supplementary Fig. 6a, only #1, #4, and #5 met economic viability, supporting the suggestion that the optimization procedure presented in Fig. 2a works efficiently by screening out economically viable process configurations beforehand. Clusters #1 and #4 share the same optimal process configuration (see Supplementary Fig. 6a), and the optimal configuration contains all the advantageous characteristics to reduce the operational cost (the use of reactive distillation and column sequencing to separate Me-TFA first). Cluster #4 was a collection of the originally selected optimization variables from the pre-screening procedure. The variables in cluster #1 can be obtained via additional continuous variable optimization, while fixing the integer variables in the same manner as those in cluster #4.

### Sensitivity analysis

Local and global sensitivity analysis (GSA) were carried out to assess the influence of uncertainties in the operating variables and TEA parameters. Fourier amplitude sensitivity testing (FAST) was used to calculate the sensitivity indices and obtain the required samples. FAST is one of the most widely used techniques for quantifying uncertainty and calculating variance-based sensitivity indices that indicate the impact of uncertainties in parameters^58,59^. Prior to analyzing the economic parameters and operating variables, sensitivity-varying reaction performance was analyzed. The GSA results indicate that the oxidation parameters exert a dominant impact.

**Fig. 3:**
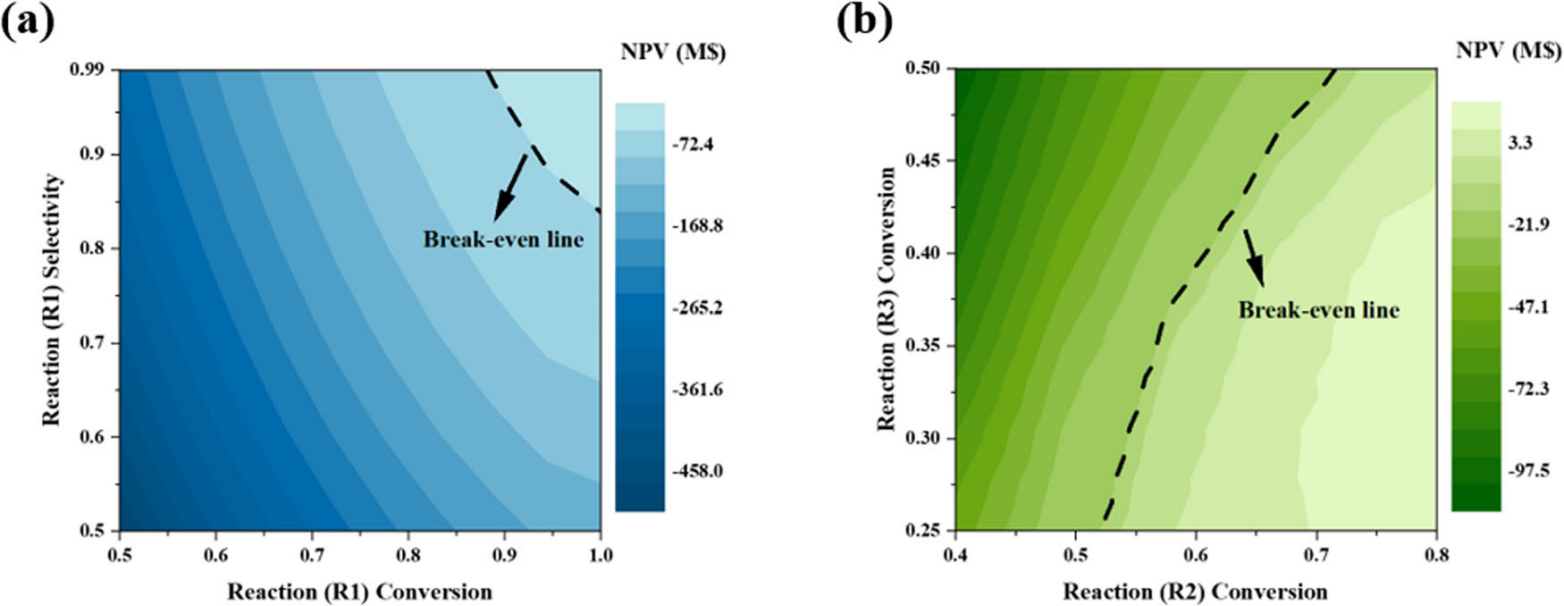
Sensitivity analysis varying reaction performance parameters. **a** Sensitivity result varying oxidation reaction sensitivity and conversion and **b** conversions of esterification and hydrolysis. The dashed line indicates the conditions where the NPV becomes zero.

Figure 3a shows the change in NPV depending on the selectivity and conversion of the oxidation reaction when the conversion of esterification and hydrolysis reactions is fixed at the optimum point obtained from the simulation. The oxidation selectivity seems to have less impact on the NPV than the conversion. This is because, as shown in Eqs. (1) and (2), the side reaction of oxidation produces commercially viable sulfuric acid which can defend the economic feasibility from the deterioration caused by the reduction of methanol production. Figure 3b shows the NPV sensitivity results obtained by varying the conversions of the esterification and hydrolysis reactions when the oxidation yield is fixed based on the above-mentioned experimental results. In contrast to hydrolysis, esterification exerts a considerable impact on NPV, but the suggested process can earn profit in most of the tested conversion ranges.

**Fig. 4:**
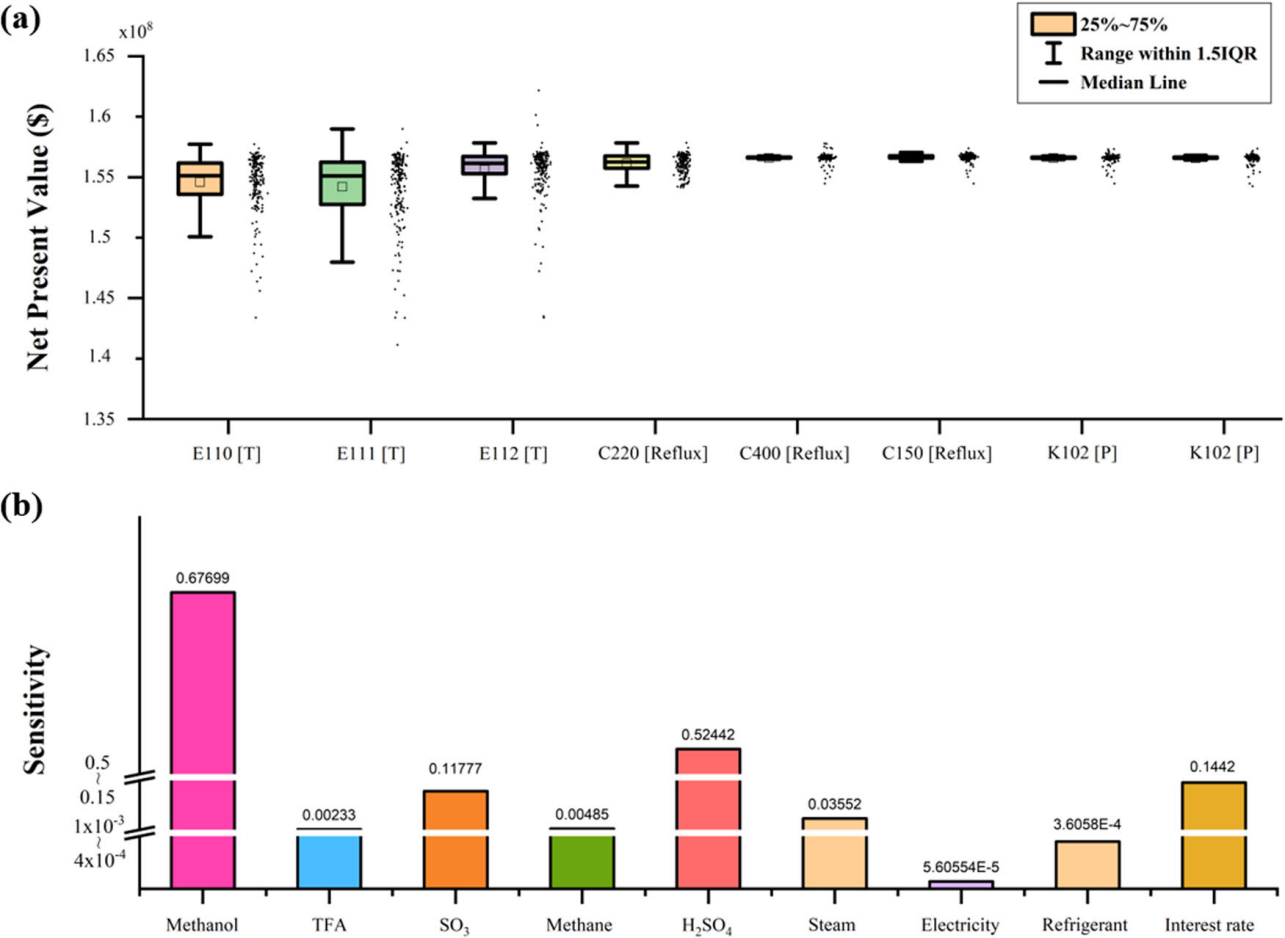
Sensitivity analysis results for operating conditions and economic parameters. **a** NPV distribution depending on varying operation conditions. Whiskers were obtained by multiplying interquartile range (IQR) by 1.5. **b** Sensitivity indices of economic parameters.

Figure 4a shows the NPV distribution based on the varying operation variables. For the eight different operational conditions, the sampling bound was set to be 80–120% of the optimal value to ensure operational feasibility and obtain accurate sensitivity indices. It turns out that the selected process configuration guarantees NPV of $1.4 × 10^8^ even with the worst operating conditions. Among the operation variables, the temperatures of the inter-stage coolers (E110, E111, and E112) had the highest influence on the NPV. The temperature changes in these units significantly affect light gases (methane and SO_2_) recovery, which in turn changes the raw material consumption. When the temperatures of the coolers are lowered by 20% of their optimally selected values, the amount of light gases decreases by 20%, while requiring an even larger amount of cooling utilities. In contrast, when the oxidation product is separated at a temperature higher than the optimum condition, the loss of the oxidation product, MBS, increases, resulting in a poor NPV. Thus, temperatures of all coolers have converged to approximately 190 °C, which was identified as optimal under the given composition of the feed.

Among the other variables, the reflux ratio of the reactive distillation column had a significant impact on the NPV. The reflux ratio has a conflicting effect on NPV, as its increase can improve the esterification conversion by extending the contact between the reactants, MBS and TFA, but simultaneously, increases the heat duty in the reboiler. To operate the reactive distillation optimally, the reflux ratio should be low as long as the column can consume most of the MBS input. As a result of the optimization, the reflux ratio that satisfies the above-mentioned condition is determined to be 5.1.

The effects of the economic parameters (feed and product prices, utility cost, and interest rate) obtained from the GSA are presented in Fig. 4b. The most influential parameters were the prices of methanol and sulfuric acid. Methane and TFA, which are designed to be recycled in the process, have little impact on process profit, implying that optimization minimizes their losses. In the case of utilities, the sensitivity index of the steam cost shows the highest value; however, the overall influence of utilities on the NPV is incomparable to that of material prices. Although the suggested process requires intensive energy input for product separation and feed recycling, the heat network selected by superstructure optimization efficiently reduces heat wastage. This means that the methanol-sulfuric acid co-production process can secure economic feasibility regardless of an unexpected increase in energy use owing to the uncertainty existing in the suggested design.

In addition to the sensitivity of the operating conditions and economic parameters, a sensitivity of the economics of the process as a function of process scale was performed. To utilize methane from small-scale facilities, the conversion process should be able to overcome the issue of economies of scale as production costs increase with diminution in scale^60^. Supplementary Fig. 7 shows a linear relationship between production capacity and NPV, showing the maximum profit when the process meets the commercial scale (generally 12,500 kg hr^−1^ = 100,000 tons yr^−1^). The process can be economically viable even at a production capacity of 2000 kg yr^−1^, which implies this process can be applied to small-scale gas fields. Detailed graphical result is provided in the Supplementary Fig. 7.

### Mitigation of carbon emission

**Fig. 5:**
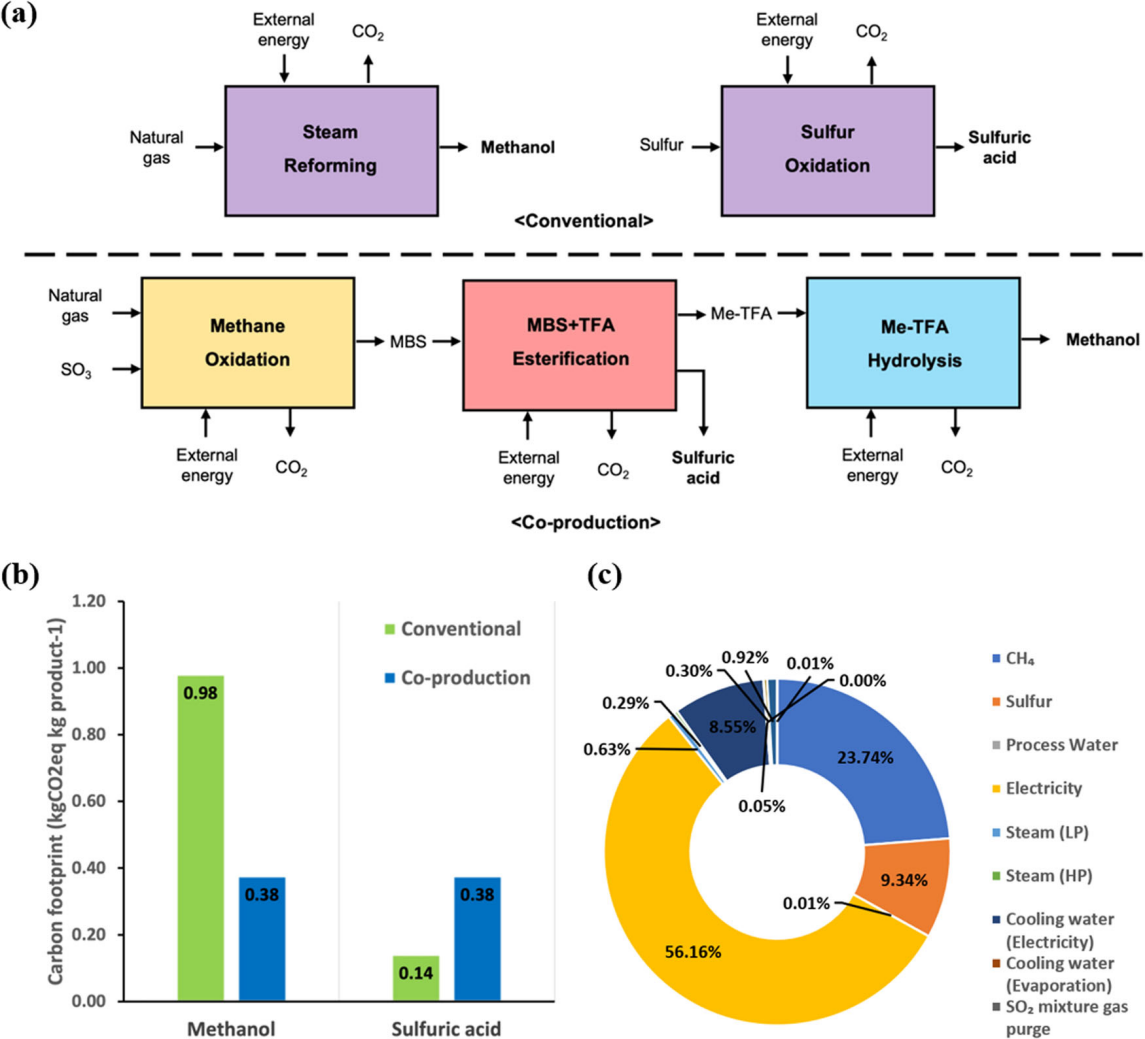
Carbon emission analysis result. **a** CO_2_ emission source breakdown. **b** Carbon footprint result. **c** Source of carbon emissions.

To analyze the global warming impact of the suggested process, the carbon footprint (CFP) was estimated and compared with that of a conventional process. As the suggested process produces methanol and sulfuric acid simultaneously, the CFP is allocated for both products according to the production rate. The CFP of each product was compared with that of a conventional process, steam methane reforming, and sulfur oxidation, as presented in Fig. 5a.

According to the Ecoinvent 3.71 database^61^, the CFP of methanol and sulfuric acid are 0.98 kgCO_2_eq kg^−1^ and 0.14 kgCO_2_eq kg^−1^, respectively. The CFP allocation results for the proposed process are shown in Fig. 5b. With respect to methanol, the suggested co-production process can significantly reduce carbon emissions. However, a relatively large amount of carbon emission is estimated in sulfuric acid production, which is mainly due to energy-intensive oxidation catalyst recycling which requires the evaporation of sulfuric acid. However, with respect to methanol, the CO_2_ emission is reduced by 0.6 kgCO_2_eq kg^−1^ methanol production. Furthermore, considering the global warming potential of methane (25 kgCO_2_eq kg^−1^), practical application of the suggested process to gas fields of which the scale is not available to conventional processes would contribute further mitigation carbon emissions. When the same amounts of both products are produced, co-production processes emit only 68% of CO_2_ as compared with the conventional process. More positive scenarios by adopting renewable energy are also presented in Supplementary Figs. 9–10.

Figure 5c shows the ratio of the emission factors of the CO_2_ produced by the raw materials and utilities used in the process and the by-products emitted. The emission factor can be calculated as the product of the inherent emission factor of each material, utility and by-product and the amount consumed or emitted to produce equivalent amount of methanol. As shown in Fig. 5c, the main source of carbon emissions is the use of electricity to generate steam energy and to operate distillation and recycling units, accounting for 56% of CO_2_ emissions. Excessive energy use is inevitable as the suggested process in the distillation of sulfuric acid which is conventionally supplied by exothermic reactions using sulfur and air.

Other than methane oxidation, CO_2_ hydrogenation can be viewed as a competitive alternative for methanol production when considering carbon emissions. In a comprehensive analysis by Rumayor et al., the CFP of methanol produced from thermochemical CO_2_ hydrogenation—with the assumed hydrogen source being water electrolysis—can be reduced to 0.23 kgCO_2_eq kg^−1^ due to the carbon negative impact of CO_2_ utilization^62^. However, given that CO_2_ hydrogenation requires 3 moles of hydrogen for every mole of methanol, the CFP could exceed 1 kgCO_2_eq kg^−1^, depending on the hydrogen source^45^. If hydrogen derived from methane reforming is used in CO_2_ hydrogenation, the CFP of methanol would be approximately 2 kgCO_2_eq kg^−1^ ^63^. For methanol production via the electroreduction of CO_2_, the CFP with current technology is even higher because of the low CO_2_ conversion, which results in high energy consumption for methanol purification^62^. This suggests that CO_2_ utilization in methanol production effectively reduces CO_2_ emissions only when green hydrogen is available and reaction performance is significantly improved. When utilizing natural gas, the proposed process can be environmentally competitive.

## Discussion

The process of co-producing methanol and sulfuric acid through direct conversion of methane reactions was designed and optimized in this study. Experiments were conducted for the methane oxidation, MBS conversion, and Me-TFA hydrolysis reactions, and the use of the experimental data in modeling the process improved the reliability of the optimization results. The proposed hybrid optimization procedure marginalizing the integer variables in the subsequent continuous variable optimization step enables the efficient identification of the optimal process design. Superstructure optimization determined the reactive distillation column used for the esterification reaction of MBS and TFA. This improves the esterification conversion from 30% to 99% of the MBS feed by simultaneously separating the reaction product Me-TFA and thereby moving the reaction equilibrium forward. Including the adoption of the reactive distillation column, the determinations by optimization enable finding the economically feasible process design retrieving the CAPEX within three project years, when the process aims to produce 100,000 tons yr^−1^ of methanol. According to the analysis results, the proposed process can reduce the levelized production cost of methanol to $203 ton^−1^ through co-production with sulfuric acid, which means that it can significantly reduce the current price of methanol production. The proposed process is attractive because its production capacity can be as small as 2000 kg hr^−1^ of methanol while meeting economic viability, meaning that it should be implemented to produce an additional profit and reduce carbon emissions by converting the methane currently flared from small gas fields into methanol.

## Methods

### Materials

All chemicals were prepared as analytical reagent grade and used without further purification. Oleum (20 wt% SO_3_), methanesulfonic acid, methyl trifluoroacetate, and trifluoroacetic acid-d 99.5 atom% D were purchased from Sigma Aldrich Co. and trifluoroacetic acid was purchased from Sejinci Co. Pt-black (surface area: 27 m^2^ g^−1^) was obtained from Alfa Aesar Co.. High-purity methane gas containing 1% of argon was supplied from Shinyang Gas Co.

### Pt black-catalyzed methane oxidation reaction

The partial methane oxidation reaction using Pt-black as a catalyst was carried out in a stainless-steel reactor (SS 316) equipped with a glass liner, thermocouple, pressure gauge and thermal jacket. In the reactor prepared for the methane oxidation, Pt-black catalyst and 30 g of 20 wt% oleum were introduced into the reactor and pressurized with 25 bar of CH_4_ at room temperature. The reactor was subsequently heated to 180 °C and stirred (800 rpm) for 3 h. When the oxidation reaction is finished, the reactor was removed from the heating jacket and placed into a water bath to be cooled down (Supplementary Fig. 1).

After the reaction, the liquid product was analyzed using 1H NMR (400 MHz, Varian) to estimate the obtained amount of MBS. To detect CO_2_ gas produced in the reactor, the gas product was collected in a plastic gas bag and analyzed using GC-MS (HP 6890 GC with a 5973 MSD) equipped with capillary column (Poraplot Q 30 m × 25 um). Argon gas which was included in methane (1%) was used as a reference to measure the concentration of CO_2_ in the gas mixture. The yield and selectivity were determined as follows.

$${{{{{\rm{Yield}}}}}}({{{{{\rm{MBS}}}}}}, \, \% ) = 100 \times \frac{{{{{{\rm{MBS}}}}}}\,{{{{{\rm{produced}}}}}}\,({{{{{\rm{mmol}}}}}})}{{{{{{\rm{SO}}}}}}_{{3}} \, {{{{{\rm{used}}}}}}\,({{{{{\rm{mmol}}}}}}) / 2}$$

$${{{{{\rm{Selectivity}}}}}}({{{{{\rm{MBS}}}}}} \, {{{{{\rm{or}}}}}} \, {{{{{\rm{CO}}}}}}_{2}, \, \% ) \\ = 100 \times \frac{{{{{{\rm{MBS}}}}}} \, {{{{{\rm{or}}}}}} \, {{{{{\rm{CO}}}}}}_{2}, \, {{{{{\rm{produced}}}}}} \,({{{{{\rm{mmol}}}}}})} {{{{{{\rm{MBS}}}}}} \, {{{{{\rm{produced}}}}}} ({{{{{\rm{mmol}}}}}}) + {{{{{\rm{CO}}}}}}_{2}\,{{{{{\rm{produced}}}}}}\,({{{{{\rm{mmol}}}}}})}$$

$${{{{{\rm{Conversion}}}}}}({{{{{\rm{CH}}}}}}_{4}, \, \% ) = 100 \times \frac{{{{{{\rm{MBS}}}}}}+{{{{{\rm{CO}}}}}}_{{2}},\,{{{{{\rm{produced}}}}}} \, ({{{{{\rm{mmol}}}}}})}{{{{{{\rm{SO}}}}}}_{3}\,{{{{{\rm{used}}}}}} \, ({{{{{\rm{mmol}}}}}}) / 2}$$

### Esterification reaction of MBS and TFA

In preparation for the esterification experiment, 0.5 g of the liquid product from the oxidation reaction containing MBS (0.54 mmol) was mixed with 0.3 g of the reference solution (5% of methanesulfonic acid as an external standard in CF_3_COOD). The concentrations of MBS and Me-TFA were measured using the 1H NMR spectroscopy (Supplementary Fig. 4). Based on the measured amount of MBS and Me-TFA, H_2_SO_4_ and TFA concentration could be determined.

### Hydrolysis reaction of Me-TFA and water

3 g of Me-TFA (0.0234 mmol) was hydrolyzed with 0.84 g of water (0.0469 mmol) within 1 h under vigorous stirring in the glass pressure tube (Ace glass, max. 150 psig) which was placed and heated in the oil bath. The correlation of the produced hydrolysis products (methanol, TFA, dimethyl ether) and the starting reagent (Me-TFA) were measured at 20 °C–150 °C using Gas chromatography (7890 A, FID, Agilent Technologies).

### Economic analysis

The net present value (NPV) is adopted as the objective function of optimization as it can provide an insightful evaluation of the suggested process in terms of economic feasibility by analyzing cash flows over the project years. The formulation of the NPV is expressed by Eq. (9).

9 $$\min {NPV}\left(x,y\right)= \mathop{\sum }\limits_{t=3}^{15}\frac{({Revenue}-{OPEX}(x,y))}{{(1+r)}^{t}} \\ - \mathop{\sum }\limits_{t=1}^{2}\frac{{CAPEX}(x,y)\times r}{{(1+r)}^{t}-1}$$

where *x* and *y* represent the operating and design variables of the process, respectively. The NPV considers a total of 15 years of projects and two years of construction. The depreciation at interest rate *r* is considered for the NPV calculation. The process design and NPV calculation were based on a methanol production scale of 100,000 tons yr^−1^. Operating expenditure (OPEX), capital expenditure (CAPEX), and revenue were calculated based on the simulation results from Aspen Plus. The detailed procedure for the NPV calculation is provided in the Supplementary Methods.

### Supplementary information

Supplementary Information

## Data Availability

The additional data supporting the findings of this study are available within the article and its Supplementary Information. Detailed experimental results are available at Supplementary Figs. 1–5 and Supplementary Tables 1–4. Analyse results of optimal and suboptimal processes are available at Supplementary Figs. 6–11 and Supplementary Tables 5–9. Additional data is available from the corresponding author upon reasonable request.
