# Supplementary material for: Economically viable co-production of methanol and sulfuric acid via direct methane oxidation
Source: Commun Chem. 2023 Dec 20;6:282. doi: 10.1038/s42004-023-01080-4 (PMC10733281; doi:10.1038/s42004-023-01080-4)
Supplement: Supplementary file 1 — Supplementary Information [file 42004_2023_1080_MOESM1_ESM.pdf]

Supplementary Information for

Economically viable co-production of methanol and sulphuric acid via direct methane oxidation

Jaehyung Im[1,2,‡], Seok-Hyeon Cheong[1,3,‡], Huyen Tran Dang[1,3,‡], Nak-Kyoon Kim[4], Sungwon Hwang[5], Ki Bong Lee[2], Kyeongsu Kim[1,*], Hyunjoo Lee[1,3,*], Ung Lee[1,*,†]

[1] Clean Energy Research Center, Korea Institute of Science and Technology (KIST), 02792 Seoul, Republic of Korea

[2] Department of Chemical and Biological Engineering, Korea University, Seoul, 02841, Republic of Korea

[3] Division of Energy & Environmental Technology, KIST School, University of Science and Technology, 02792 Seoul, Republic of Korea

[3] Advanced Analysis Center, Korea Institute of Science and Technology, Seoul 02792, Republic of Korea

[5] Department of Chemical Engineering, Inha University, Incheon, Republic of Korea

*Corresponding author.

E-mail address: kyeongsu@kist.re.kr (Kyeongsu Kim), hjlee@kist.re.kr (Hyunjoo Lee), ulee@kist.re.kr (Ung Lee)

† Request for materials should be sent to this author

‡ These authors contribute equally to this study

Experiment

Pt black-catalyzed methane oxidation reaction

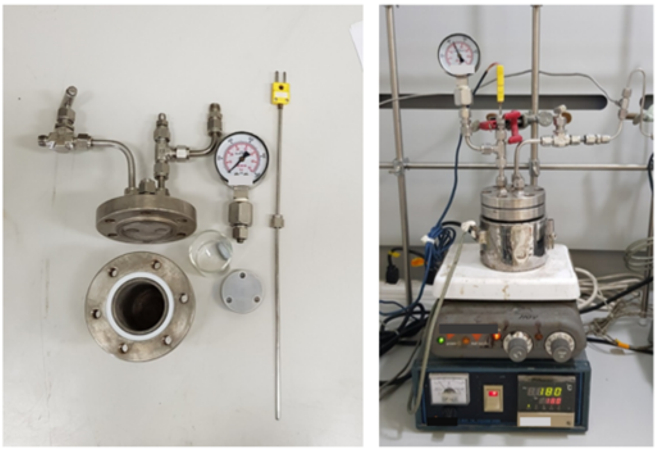

Supplementary Figure 1. Photos of pressure reactor and reaction system for the methane oxidation.

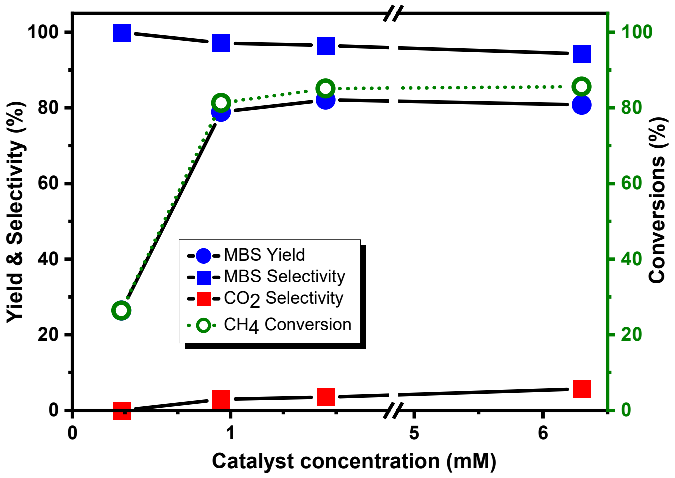

Supplementary Figure 2. Effect of Pt-black concentrations on the Pt-black catalyzed methane oxidation to methyl bisulfate (MBS). Conditions: oleum (20 wt% SO_3), 25 bar of CH_4, 180 °C, 3 h. [1]

The repeated experiment for optimization was guided by the Bayesian optimization algorithm to efficiently find the optimal reaction temperature and oleum concentration [2-4].

Supplementary Table 1. Optimization results methane oxidation reaction.

Entry	Temperature (°C)	SO_3 (wt%)	Turnover number
1	180.0	0.0	0.0
2	150.0	20.0	139.7
3	239.0	38.8	744.1
4	180.0	65.0	813.6
5	168.8	36.5	1027.4
6	220.0	20.0	1082.8
7	200.0	20.0	1168.3
8	180.0	20.0	1199.5
9	202.3	38.0	1564.9
10	214.6	49.4	1466.3
11	200.7	27.9	1578.2
12	195.0	36.4	1602.3
13	186.2	30.8	1153.1
14	210.9	43.0	1313.2
15	233.5	53.8	1218.5
16	223.5	58.0	1307.2

As listed in Supplementary Table 1, the best turnover number found was 1,602.3 under the reaction temperature of 195 °C and 36.4 wt% of oleum. The experimental optimization results were adopted for simulation of the oxidation process.

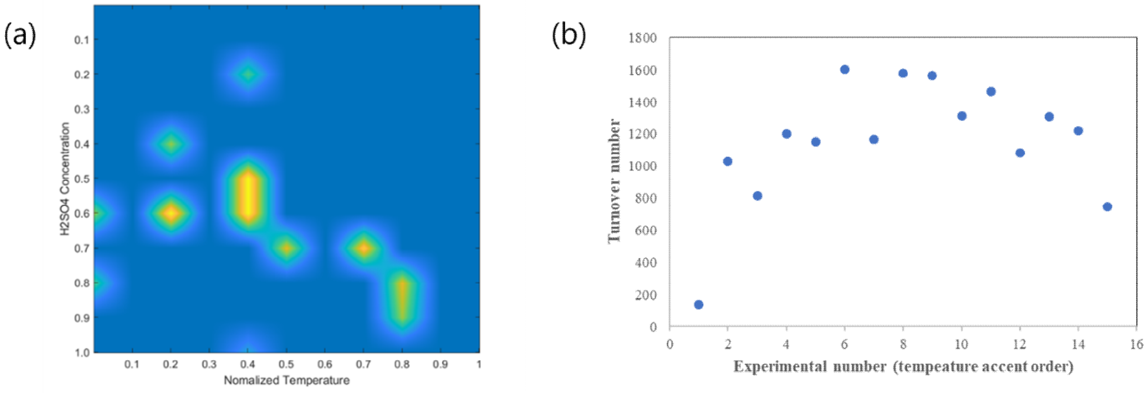

Supplementary Figure 3. (a) Search domain of the methane oxidation reaction during the Gaussian process Bayesian optimization. (b) TON changes in methane oxidation reaction according to the temperature increases.

Supplementary Note 1.

Various cutting-edge technologies producing methane to methanol are presented in Supplementary Table 2. We identified that gas-phase reactions, in-situ generation of H_2O_2 using H_2 and O_2, and plasma usage without catalysts displayed methane oxidation reactivity levels, with turnover frequencies (TOF) ranging from 0.003 to 50. These reactivity levels are markedly lower to the performance exhibited by the Pt catalyst employed in our research, which demonstrated a TOF ranging from 1000 to 7000.

Supplementary Table 2. Comparison of state-of-the-art methane oxidation system for methanol synthesis.

Reaction type	Cat.	Temp. (°C)	Oxidant	Catalyst Activity		Ref.
				umol/g_{cat}/h	TOF	
Gas Phase	Cu-ZSM-5 (4 wt%)	200	O_2	32.8	0.052	JACS, 2005, 127, 1394
	Cu-mordenite	200	H_2O		0.408	Science, 2017, 356, 523
	Cu-mordenite	200	H_2O		0.632	Angew. Chem. Int. Ed., 2018, 130, 9044
	Cu-mordenite (2.3wt%)	200	O_2		0.078	JACS, 2018, 140, 15270
	Cu-SSZ-13 (4 wt%)	200	O_2	125	0.2	JACS, 2017, 139, 14961
	Cu-mordenite	200	O_2		0.020	ACS Catal., 2021, 11, 4973
	Cu-SSZ-13 (2 wt%)	200	NOx	12	0.038	Fuel, 2022, 309, 122178
	Cu-mordenite (1.8 wt%)	200	O_2	40	0.145	JACS Au, 2021, 1, 1412
	Cu-SSZ-13 (2.1 wt%)	350	H_2O	189.9	0.578	Fuel, 2022, 329, 125483
Liquid Phase	5%AuPd/TiO_2	70	H_2/O_2	57.9	0.164	Angew. Chem. Int. Ed., 2013, 52, 1280

Au(3.24wt%) Pd(1.76wt%) @ZSM-5-C16	70	H_2/O_2	4580	27.7	Science, 2020, 367, 193
Pd/PMA (0.23wt%)	24	H_2/O_2	67.4	3.12	Nature Catalysis, 2023, 1-11
Pd(0.28wt%)-Fe(0.56wt%) /ZSM-5	30	H_2/O_2	200[a]	7.6	ChemCatChem, 2023, 15, e202201630
Rh/TiO_2[b] (1.0wt%)	150	H_2/O_2	5200	53	Angew. Chem. Int. Ed., 2022, 61, 18, e202201540

[a] Calculated TOF from umol/g_{cat}/h and Cu wt%
[b] reduce water with CO to produce H_2

Esterification reaction of MBS and TFA

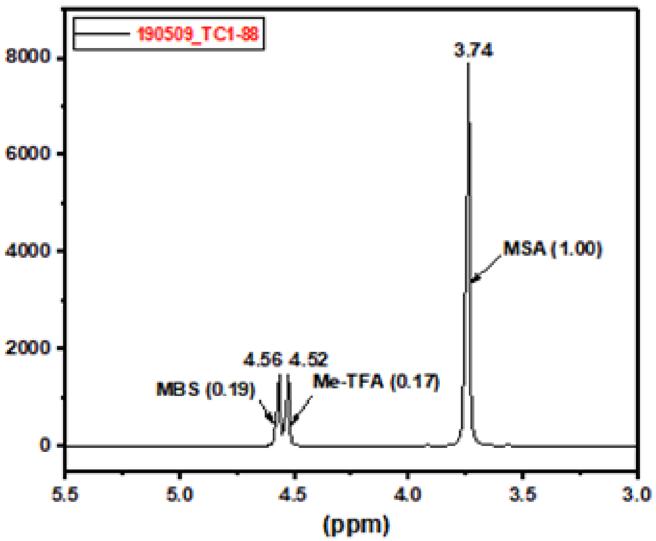

Supplementary Figure 4. 1H NMR of the mixture containing methybisulfate (MBS) and methy trifluoroacetate (Me-TFA).

Supplementary Table 3. Transesterification reaction result in the batch reactor.

Entry	Temperature (°C)	Reaction Time (h)	MBS Conversion (%)
1		0	0
2		1	11.25
3		2	16.19
4		4	26.08
5		6	34.51
6	25	8	39.53
7		10	45.45
8		12	50.78
9		24	64.78
10		36	70.28
11		48	71.1
12		0	0
13		1	30.92
14		2	41.96
15		4	57.97
16	40	6	65.96
17		8	68.45
18		10	70.39
19		12	72.04
20		24	72.58
21		0	0
22		1	51.97
23		2	57.54
24		4	62.86
25	60	6	64.54
26		8	66.21
27		10	66.77
28		12	67.33
29		24	68.17

Supplementary Note 2.

The abovementioned transesterification reaction can be accelerated through the incorporation of a reactive distillation process, which concurrently facilitates the production and separation of the derivatives. Given that methyl Me-TFA undergoes simultaneous evaporation upon its formation during the reactive distillation, the transesterification reaction is further driven towards the product side, enhancing the overall yield. Supplementary Table 4 compiles the outcomes of the transesterification reaction within the reactive distillation column. The data presented therein affirm an improvement in Me-TFA yield, elevating it to 86%.

Supplementary Table 4. Experimental results from the continuous operation of reactive distillation recovering methyl trifluoroacetate (Me-TFA).

Exp.	TFA in (mol)	MBS in (mol)	Me-TFA Product (mol)	Yield (%)
**1**	4.5	3.39	1.95	57.4
**2**	4.39	3.31	1.69	51.1
**3**	5.86	2.48	1.95	78.5
**4**	15.7	11.9	7.82	65.9
**5**	14.9	11	7.21	65.8
**6**	14	10.1	7.07	69.8
**7**	11.7	8.19	6.11	74.6
**8**	13.4	9.77	7.42	76
**9**	7.35	5.4	4.3	79.7
**10**	13.5	9.92	8.52	85.8
**11**	10.3	7.17	6.06	84.5

Hydrolysis reaction of Me-TFA and water

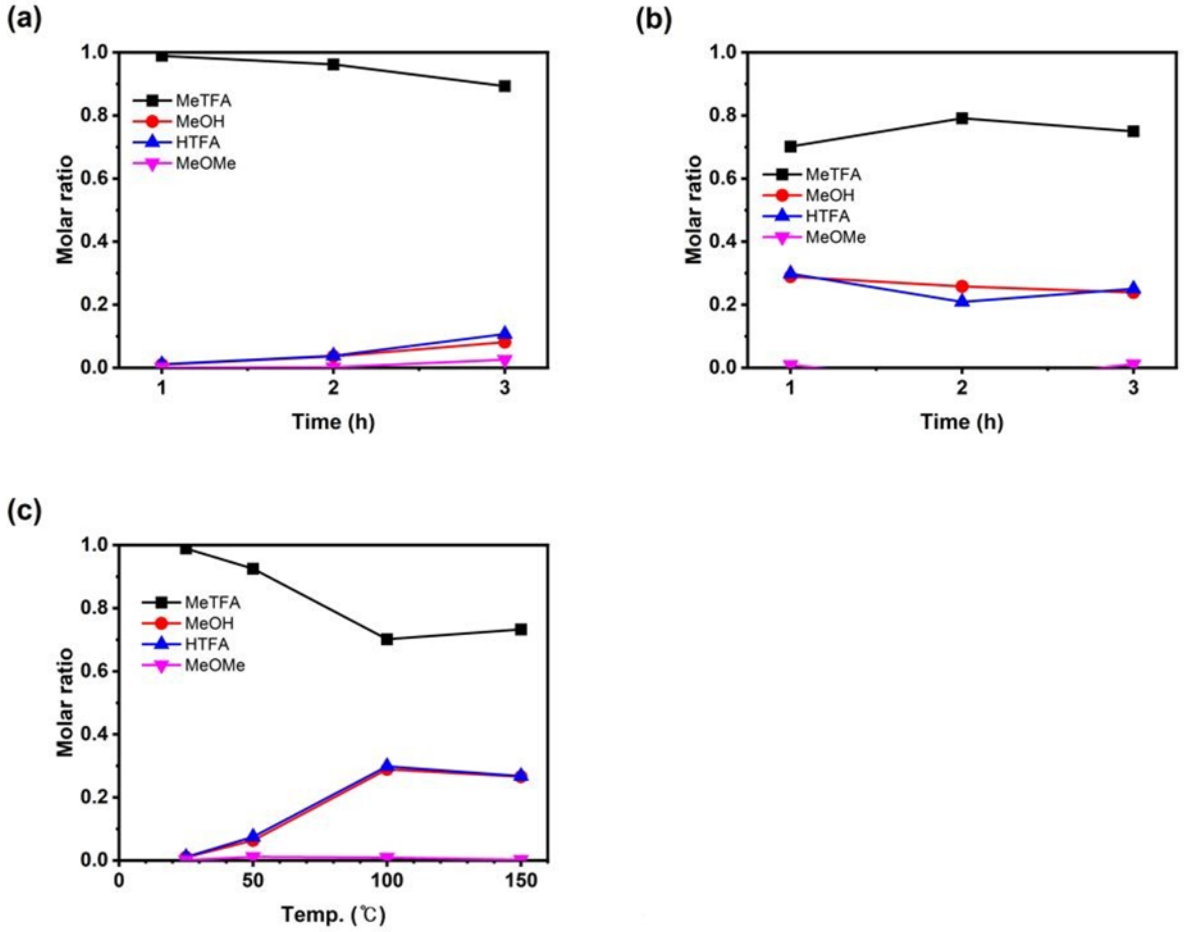

Supplementary Figure 5. (a) Time scanning experiments on methyl trifluoroacetate hydrolysis reaction. Conditions: Molar ratio of Me-TFA : H_2O = 1 : 1 , 25 °C. (b) Time scanning experiments on methyl trifluoroacetate hydrolysis reaction. Conditions: Molar ratio of Me-TFA : H_2O = 1 : 1 , 100 °C. (c) Temperature scanning experiments on methyl trifluoroacetate hydrolysis reaction. Conditions: Molar ratio of Me-TFA : H_2O = 1 : 1 , 1 h.

Analysis of suboptimal process configurations

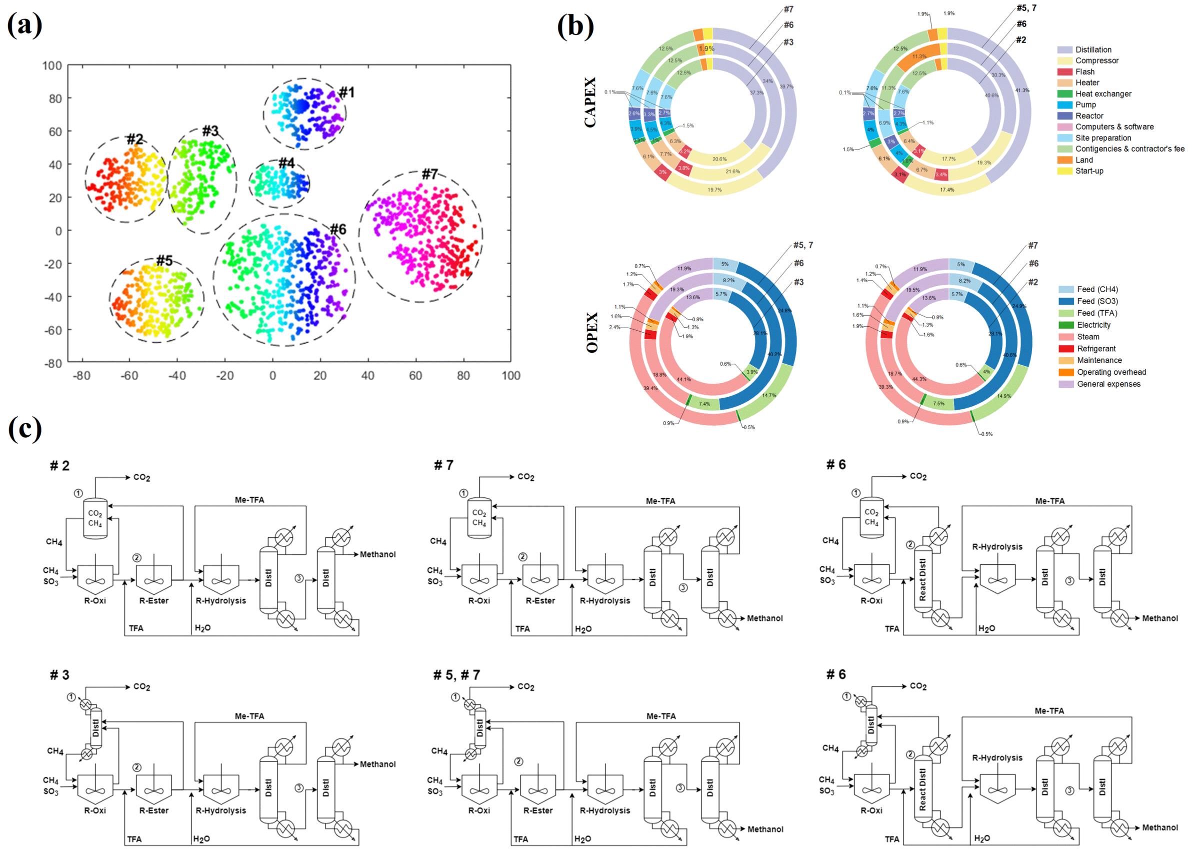

Supplementary Figure 6. Analysis results for optimum process design; (a) The *t*-SNE diagram of optimization variables and (b) cost breakdown for each variable cluster. (c) Simplified process configuration corresponding to each cluster. Note that each cluster can have more than one configuration.

Supplementary Figure 6 illustrates the *t*-distributed stochastic neighbor embedding (*t*-SNE) results for the collected data obtained during optimization.

Economics depending on methanol production scale.

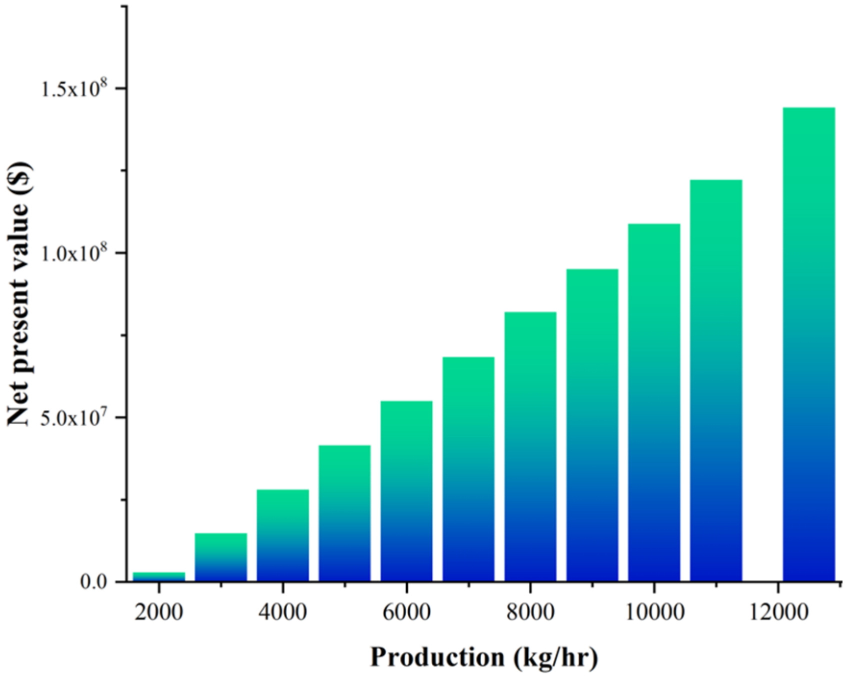

Supplementary Figure 7. NPV of the optimal process depending on the methanol production capacity.

The minimum production capacity of methanol that can earn profit was 2,000 kg hr^{-1} which requires 1,000 kg hr^{-1} consumption of methane; thus, the suggested process can be applicable to small- or medium-sized gas fields.

Superstructure design

(a)

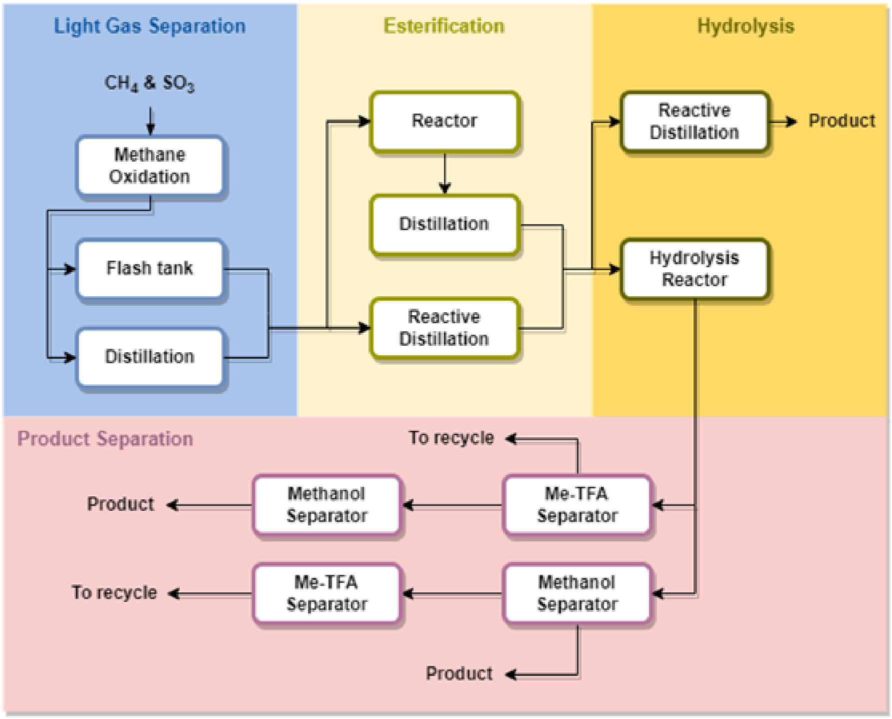

(b)

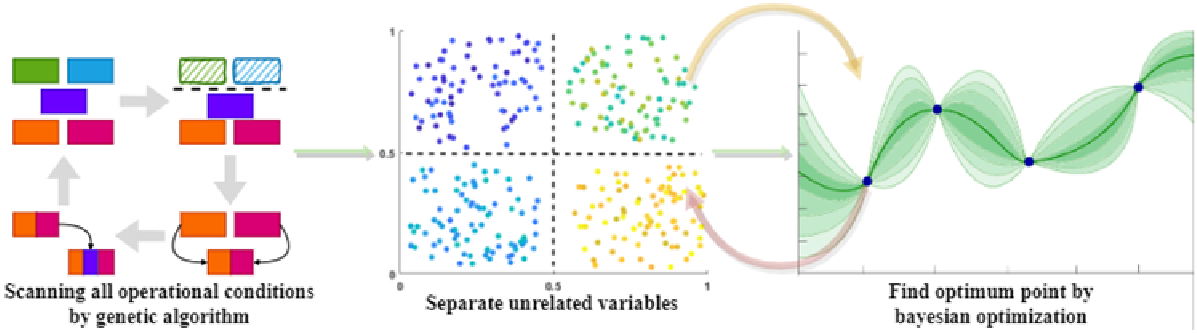

Supplementary Figure 8: Superstructure optimization scheme. (a) Overview of proposed superstructure presenting possible process options. (b) Procedure of superstructure optimization

Supplementary Figure 8 shows the superstructure and optimization method considered in this study. The upper color shaded blocks in the Supplementary Figure 8 (a) represent the oxidation, heat integration, esterification, and hydrolysis sections respectively.

Carbon foot prints with renewable energy sources

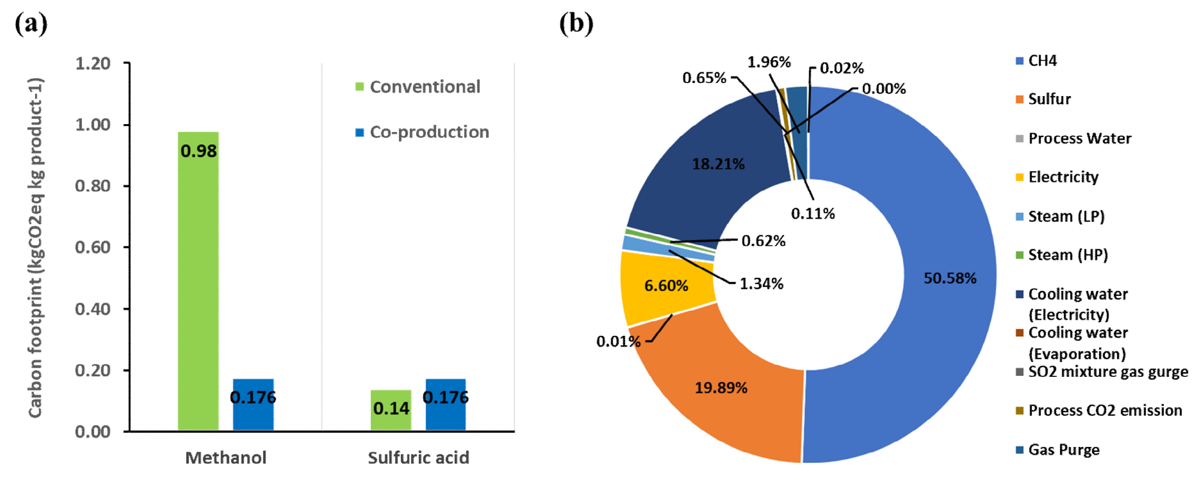

Supplementary Figure 9: Carbon emission analysis result when electricity is solemnly supplied by wind. (a) Carbon footprint result. (b) Source of carbon emissions.

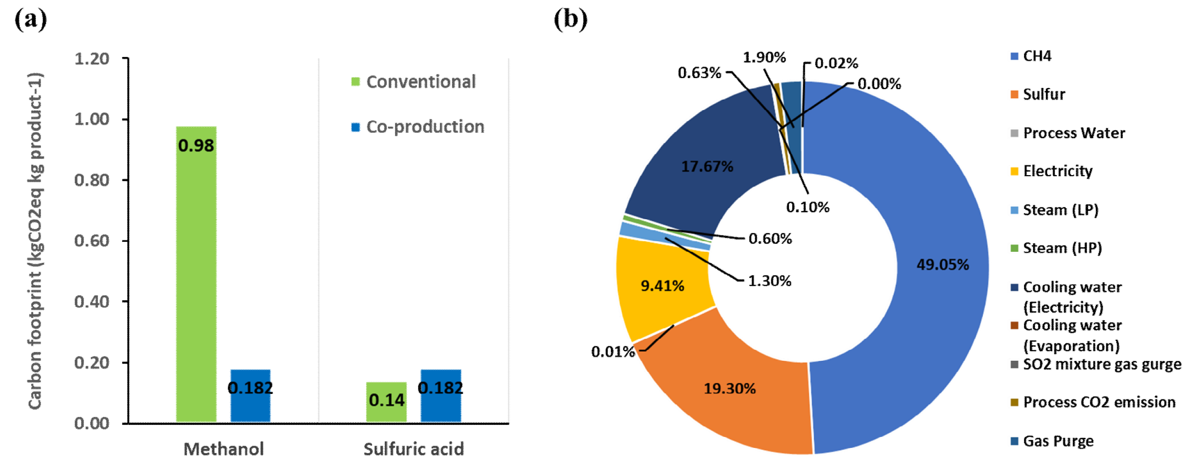

Supplementary Figure 10: Carbon emission analysis result when electricity is solemnly supplied by solar energy. (a) Carbon footprint result. (b) Source of carbon emissions.

Process description

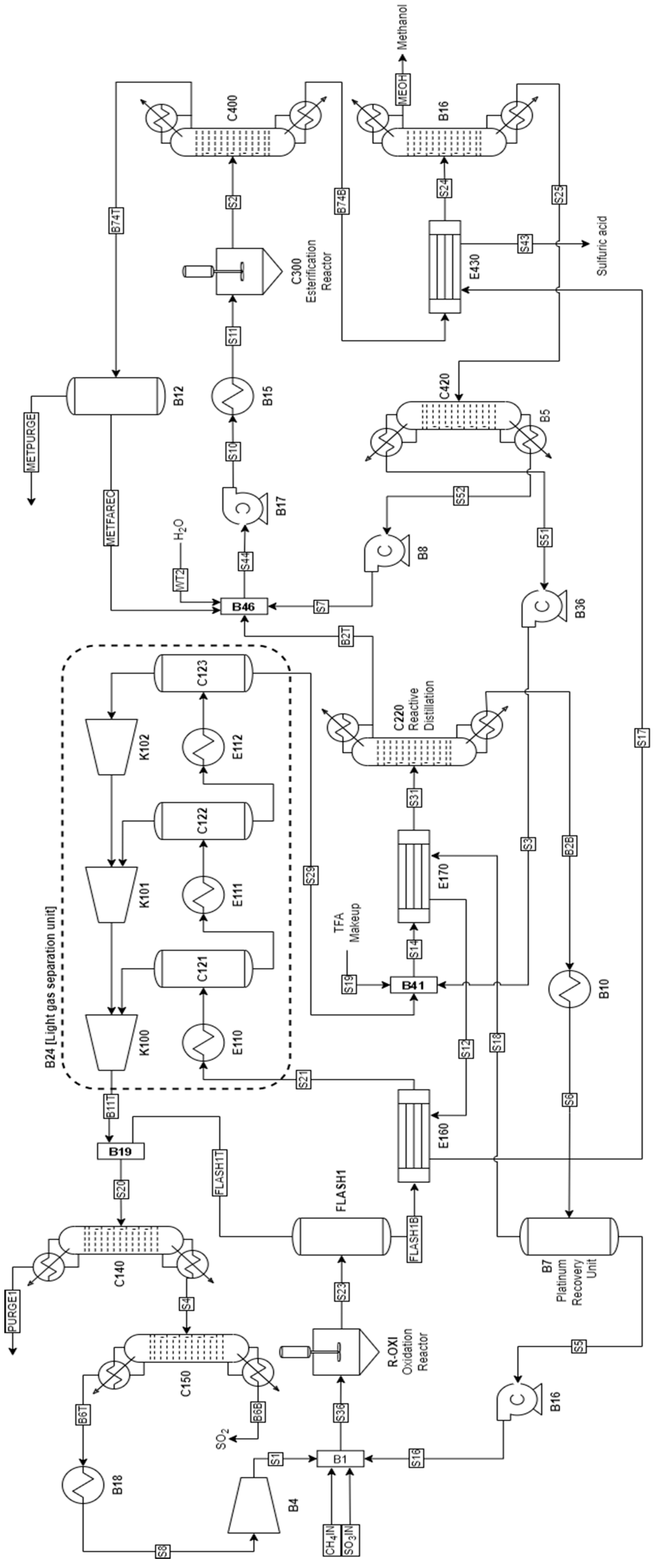

Supplementary Figure 11. Process flow diagram determined by superstructure optimization.

Supplementary Table 5. Process unit description.

Unit		Description
Reactor	R-OXI & FLASH1	To simulate the mixed phase partial oxidation reaction of gas state methane and liquid state 20 wt% oleum, R-Stoic reactor and flash unit which are embedded in Aspen Plus were used. Referring to the experimental result, pressure and temperature were set to be 180 °C and 25 bar, respectively. In this unit, 77 wt% of methane gas is converted into methyl bisulfate and SO_2 accounts for 75 wt% of the gas phase product under the reaction conditions, which in turn, is fed to the gas separation unit, FLASH1.
	C300	C300 is the hydrolysis reactor unit simulating the reaction of Me-TFA and H_2O to produce TFA and methanol. In order to calculate correlation between the K_{eq} and the reaction temperature, built in K_{eq} expression as $\ln K_{eq} = 31.903 \ln T - 189.05$ was used based on the experimental data shown in Fig. 1 (e). 46 % of the Me-TFA is reacted with water at 100 °C and 3 bar.
Separator	C140	C140 is the CO_2 separation column, which is designed to remove 50 % of CO_2 in the column feed stream. Since the recycle stream contains 0.02 % of CO_2, CO_2 should be removed in order to prevent from the accumulation of CO_2 in the recycle stream.
	C150	C150 is the SO_2 separation column, of which feed stream contains 75 % SO_2 and 24 % methane. In order to reduce the raw material cost, methane gas is separated and recycled, and 99.5 % purity of SO_2 gas is produced. The reflux ratio and the boil-up ratio are set as 5.15 and 0.41.
	C220	C220 is the reactive distillation column where esterification reaction between MBS and TFA occurs. The pressure was set to 1 bar in order to lower the separation temperature of the H_2SO_4 solution. K_{eq} parameter of H_2SO_4 decomposition reaction is calculated by Gibbs energy equilibrium, and K_{eq} built-in expression of the esterification reaction is used as $\ln K_{eq} = \frac{265.85}{T} + 1.012,$ which is obtained by regression of the experimental data shown in Fig. 1 (d). The reflux ratio and the boil-up ratio are set as 7 and 0.63. The number of stages is designed to be 40.
	C400	C400 is the Me-TFA separation column separating 36.3 % of Me-TFA in the feed stream. Since a trace amount (< 1 %) of SO_2 is distilled with the Me-TFA, 2.2 % of the recycle stream has to be purged in order to prevent the accumulation of SO_2. The separated Me-TFA is then recycled back to the hydrolysis reactor, C300. The reflux ratio and the boil-up ratio are set as 3.98 and 0.96. The number of stages is designed to be 50.
	C420	C420 separates TFA from the other components (mainly water), which are produced from the hydrolysis reaction. As the TFA and water form an azeotropic mixture, C420 is designed to be operate at vacuum condition (0.1 bar). The distillate consisting of 99.7 % TFA is recycled to the esterification reaction. The TFA loss is compensated by the TFA make-up stream, S19. The reflux ratio and the boil-up ratio are set as 5 and 2.17. The number of stages is designed to be 50.

	**B16**	B16 is the methanol separation column aiming at producing 12,500 kg hr^{-1} of methanol with 99 wt% purity. Design specification was set to secure the 99 wt% of the methanol purity and the corresponding minimum required reflux ratio was calculated as 7.06. The boil-up ratio is set as 2.24. The number of stages is designed to be 80.
	**B24**	B24 is the light gas separation unit consisting of 3 heaters, flash drums, and compressors. B24 separates CO_2, CH_4, SO_3, and SO_2 accounting for 6.7 wt% of the B24 feed stream. It was confirmed that 94 % of the fed light gas components could be separated by multi-stage flash separation. The optimum compression ratio of the three compressors and the outlet temperatures of the heaters were determined by continuous variable optimization. In the order of the compressors, K100, K101, and K102, the discharge pressures are determined as 23 bar, 9 bar, and 3 bar. The outlet temperature is set as 200 °C for all the heaters, E110, E111, and E112.
**Heater & Heat Exchanger**	**E170**<br>**E160**<br>**E430**	The latent heat of the H_2SO_4 vapor stream is exchanged with the esterification feed stream preheater E170. Condensed H_2SO_4 stream provides heat duty to light gas separation unit (E160) and methanol separation column (E430) sequentially.
	**B10**	B10 is the heater for the platinum recovery unit. B10 is designed to evaporate H_2SO_4 solution by the equivalent amount that is formed in the CH_4 oxidation reactor to maintain overall H_2SO_4 concentration (According to the oxidation experiment result, the weight concentration of SO_3 should be fixed at 36.4 wt% to attain the best reaction performance. This concentration is controlled by the amount of H_2SO_4 recycled). For this reason, 12.5 wt% of the B10 feed stream should be evaporated.
	**B15**	B15 is a preheater for the hydrolysis reactor (C300), and outlet specification was set as 100 °C, which is the optimum hydrolysis temperature to produce the methanol from Me-TFA.
	**B18**	B18 is a preheater for the methane compressor, which prevents methane gas from liquefied during compression.

Mass balance table

Supplementary Table 6. Mass balance table obtained from optimal process design.

Stream	T (°C)	P (bar)	Vapor Frac.	Liquid Frac.	Mole Flow (kmol hr^{-1})	Mass Flow (kg hr^{-1})	Mass Fraction										
							O_2	CH_4	CO_2	SO_2	Me-TFA	SO_3	MeOH	TFA	H_2O	MBS	H_2SO_4
B2B	274.2	1	0.00	1.00	3098.9	303935	0.00	0.00	0.00	0.00	0.00	0.00	0.00	0.00	0.00	0.00	1.00
B2T	35.4	1	0.00	1.00	418.1	51767	0.00	0.00	0.00	0.03	0.97	0.00	0.00	0.00	0.00	0.00	0.00
B6B	75.4	23	0.00	1.00	390.6	25118	0.00	0.00	0.00	0.97	0.00	0.00	0.00	0.00	0.00	0.01	0.02
B6T	-67.9	23	1.00	0.00	117.6	1892	0.00	1.00	0.00	0.00	0.00	0.00	0.00	0.00	0.00	0.00	0.00
B11T	449.0	23	1.00	0.00	364.7	21779	0.00	0.03	0.00	0.94	0.00	0.00	0.00	0.00	0.00	0.01	0.02
B74B	82.5	1	0.00	1.00	1563.2	101438	0.00	0.00	0.00	0.00	0.00	0.00	0.12	0.52	0.08	0.27	0.00
B74T	41.7	1	1.00	0.00	477.7	59830	0.00	0.00	0.00	0.02	0.98	0.00	0.00	0.00	0.00	0.00	0.00
CH4IN	30.0	27	1.00	0.00	400.0	6417	0.00	1.00	0.00	0.00	0.00	0.00	0.00	0.00	0.00	0.00	0.00
PURGE1	376.9	23	1.00	0.00	1.3	58	0.00	0.00	1.00	0.00	0.00	0.00	0.00	0.00	0.00	0.00	0.00
FLASH1B	180.0	25	0.00	1.00	3484.2	332549	0.00	0.00	0.00	0.07	0.00	0.00	0.00	0.00	0.00	0.13	0.80
FLASH1T	180.0	25	1.00	0.00	144.9	5289	0.00	0.25	0.01	0.74	0.00	0.00	0.00	0.00	0.00	0.00	0.00
MEOH	64.2	1	0.00	1.00	391.7	12644	0.00	0.00	0.00	0.00	0.01	0.00	0.99	0.00	0.00	0.00	0.00
METFAREC	41.7	1	0.00	1.00	456.8	58490	0.00	0.00	0.00	0.00	1.00	0.00	0.00	0.00	0.00	0.00	0.00
METPURGE	41.7	1	1.00	0.00	20.9	1341	0.00	0.00	0.00	0.99	0.00	0.01	0.00	0.00	0.00	0.00	0.00
S1	117.7	27	1.00	0.00	117.6	1892	0.00	1.00	0.00	0.00	0.00	0.00	0.00	0.00	0.00	0.00	0.00
S2	100.0	3	0.04	0.96	2040.9	161269	0.00	0.00	0.00	0.01	0.36	0.00	0.08	0.33	0.05	0.17	0.00
S3	17.6	1	0.00	1.00	396.1	44816	0.00	0.00	0.00	0.00	0.00	0.00	0.00	1.00	0.00	0.00	0.00
S4	376.9	23	1.00	0.00	508.2	27010	0.00	0.07	0.00	0.90	0.00	0.00	0.00	0.00	0.00	0.01	0.02
S5	274.4	1	0.00	1.00	2708.8	265688	0.00	0.00	0.00	0.00	0.00	0.00	0.00	0.00	0.00	0.00	1.00
S6	274.4	1	0.13	0.87	3098.9	303935	0.00	0.00	0.00	0.00	0.00	0.00	0.00	0.00	0.00	0.00	1.00
S7	53.4	1	0.00	1.00	775.4	43979	0.00	0.00	0.00	0.00	0.00	0.00	0.00	0.18	0.19	0.62	0.01
S8	100.0	23	1.00	0.00	117.6	1892	0.00	1.00	0.00	0.00	0.00	0.00	0.00	0.00	0.00	0.00	0.00
S10	42.2	3	0.00	1.00	2040.9	161269	0.00	0.00	0.00	0.01	0.67	0.00	0.00	0.05	0.09	0.17	0.00
S11	100.0	3	0.11	0.89	2040.9	161269	0.00	0.00	0.00	0.01	0.67	0.00	0.00	0.05	0.09	0.17	0.00
S12	273.1	1	0.00	1.00	390.1	38247	0.00	0.00	0.00	0.00	0.00	0.00	0.00	0.00	0.00	0.00	1.00
S14	167.9	1	0.06	0.94	3516.7	355702	0.00	0.00	0.00	0.00	0.00	0.00	0.00	0.13	0.00	0.12	0.75
S16	276.4	27	0.00	1.00	2708.8	265688	0.00	0.00	0.00	0.00	0.00	0.00	0.00	0.00	0.00	0.00	1.00
S17	183.0	1	0.00	1.00	390.1	38247	0.00	0.00	0.00	0.00	0.00	0.00	0.00	0.00	0.00	0.00	1.00
S18	274.4	1	1.00	0.00	390.1	38247	0.00	0.00	0.00	0.00	0.00	0.00	0.00	0.00	0.00	0.00	1.00
S19	30.0	1	0.00	1.00	1.0	117	0.00	0.00	0.00	0.00	0.00	0.00	0.00	1.00	0.00	0.00	0.00
S20	376.9	23	1.00	0.00	509.5	27069	0.00	0.07	0.00	0.90	0.00	0.00	0.00	0.00	0.00	0.01	0.02
S21	189.1	25	0.00	1.00	3484.2	332549	0.00	0.00	0.00	0.07	0.00	0.00	0.00	0.00	0.00	0.13	0.80
S23	180.0	25	0.02	0.98	3629.1	337838	0.00	0.01	0.00	0.08	0.00	0.00	0.00	0.00	0.00	0.13	0.79
S24	84.1	1	0.05	0.95	1563.2	101438	0.00	0.00	0.00	0.00	0.00	0.00	0.12	0.52	0.08	0.27	0.00
S25	110.7	1	0.00	1.00	1171.6	88795	0.00	0.00	0.00	0.00	0.00	0.00	0.00	0.59	0.09	0.31	0.00
S29	200.0	1	0.00	1.00	3119.5	310769	0.00	0.00	0.00	0.00	0.00	0.00	0.00	0.00	0.00	0.14	0.85
S31	205.4	1	0.10	0.90	3516.7	355702	0.00	0.00	0.00	0.00	0.00	0.00	0.00	0.13	0.00	0.12	0.75
S36	169.2	27	0.06	0.94	4023.8	337838	0.00	0.02	0.00	0.00	0.00	0.19	0.00	0.00	0.00	0.00	0.79
S43	85.5	1	0.00	1.00	390.1	38247	0.00	0.00	0.00	0.00	0.00	0.00	0.00	0.00	0.00	0.00	1.00
S44	42.2	1	0.00	1.00	2040.9	161269	0.00	0.00	0.00	0.01	0.67	0.00	0.00	0.05	0.09	0.17	0.00
S51	17.4	0.1	0.00	1.00	396.1	44816	0.00	0.00	0.00	0.00	0.00	0.00	0.00	1.00	0.00	0.00	0.00
S52	53.4	0.1	0.00	1.00	775.4	43979	0.00	0.00	0.00	0.00	0.00	0.00	0.00	0.18	0.19	0.62	0.01
SO3IN	30.0	27	0.00	1.00	797.4	63840	0.00	0.00	0.00	0.00	0.00	1.00	0.00	0.00	0.00	0.00	0.00
WT2	25.0	1	0.00	1.00	390.6	7038	0.00	0.00	0.00	0.00	0.00	0.00	0.00	0.00	1.00	0.00	0.00

Supplementary Methods

Techno Economic Analysis

To calculate the net present value of the process, the market prices of the raw materials were investigated to estimate the operating expenditure (OPEX) and the capital expenditure (CAPEX). Feed cost, utility cost, and other maintenance costs are included in OPEX. Different prices are set for the steam and refrigerant utilities depending on their operating temperature range. The total capital investment (TCI) is considered in calculating cash flows regarding CAPEX, which is the sum of the total permanent investment (TPI) and the working capital investment. TPI and the total depreciable capital (TDC) are calculated according to the following equations.

TPI = Cost of land (2 % of TDC) + Cost of plant startup (2 % of TDC) + TDC

TDC = Cost of contingencies and contractor's fee (15 % of DPI) + DPI

where DPI indicates the direct permanent cost. DPI can be obtained from the total bare-module cost (TBM).

DPI = Cost of site preparation (10 % of TBM) + TBM

TBM = Bare-module costs for equipment + costs for computers, and software

The bare-module costs for equipment were calculated via Guthrie's method [5] and the required data was obtained using Aspen Plus simulation. The costs for the computers and the related software are assumed to be $20,000. Depreciation cost was considered by modified accelerated cost recovery system (MACRS) for 7 years of depreciation. Several assumptions for the cost parameters are made such as 15 years of plant life, 2 years of construction period, 15 % of nominal interest rates, 8,000 h of operating per year, and 38.9 % income tax rate to calculate net present value. The other cost parameters including material prices and utility costs are listed in Supplementary Table 7.

The OPEX is calculated by comprehensively considering factors such as operations, maintenance, operating overhead, and general expenses. The calculation procedures for operating cost factors are summarized in Supplementary Table 8. Note that the "sales" in Supplementary Table 8 indicates the annual earnings from selling the methanol and sulfuric acid products.

Operating cost calculation

Supplementary Table 7. Parameters for techno economic analysis.

Parameters	Unit	Value
Material price		
CH_4	\$ kg^{-1}	0.1312
SO_3	\$ kg^{-1}	0.13
TFA	\$ kg^{-1}	5
H_2SO_4	\$ kg^{-1}	0.27
Methanol	\$ kg^{-1}	0.45
Utility price		
Steam, 450 psig	\$ kg^{-1}	0.0145
Steam, 150 psig	\$ kg^{-1}	0.0105
Steam, 50 psig	\$ kg^{-1}	0.0066
Process water	\$ m^{-3}	0.2
Refrigeration, -150 °F	\$ GJ^{-1}	12.60
Refrigeration, -90 °F	\$ GJ^{-1}	10.30
Refrigeration, -30 °F	\$ GJ^{-1}	7.90
Refrigeration, 10 °F	\$ GJ^{-1}	5.50
Chilled water, 0 °F	\$ GJ^{-1}	4.00
Cooling water	\$ m^{-3}	0.02
Direct wages and benefit (DW&B)	\$ $operator^{-1}$ hr^{-1}	35
Number of workers	-	10
Tech assistance to manufacturing	\$ shift $operator^{-1}$ yr^{-1}	60,000
Control laboratory	\$ shift $operator^{-1}$ yr^{-1}	65,000
Operating hour	hr yr^{-1}	8,000
Economic factors		
Plant life	year	15
Construction period	year	2
Income tax	%	38.9
Interest rate	%	15
MACRS	-	7-year class

Supplementary Table 8. Operating cost factor calculation.

Cost Factor		Annual Cost ($)
Operations (labor-related) (O)		
	Direct wages and benefits (DW&B)	$ 2,800,000
	Direct salaries and benefits	15 % of DW&B
	Operating supplies and services	6 % of DW&B
	Technical assistance to manufacturing	$200,000
	Control laboratory	$216,667
Maintenance (M)		
	Wages and benefits (MW&B)	3.5 % of TDC
	Salaries and benefits	25 % of MW&B
	Materials and services	100 % of MW&B
	Maintenance overhead	5 % of MW&B
Operating overhead		
	General plant overhead	7.1 % of M&O-SW&B
	Mechanical department services	2.4 % of M&O-SW&B
	Employee relations department	5.9 % of M&O-SW&B
	Business services	7.4 % of M&O-SW&B
Property taxes and insurance		2 % of TDC
Depreciation		
	Direct plant	8 % of (TDC-1.18alloc)
	Allocated plant	6 % of 1.18alloc
General Expenses		
	Selling (or transfer) expense	3 % (1 %) of sales
	Direct research	4.8 % of sales
	Allocated research	0.5 % of sales
	Administrative expense	2.0 % of sales
	Management incentive compensation	1.25 % of sales

Supplementary Table 9. Product carbon footprint information

	Carbon Source		Relative Amount	Unit	Reference	Emission Factor	Carbon Emission
Input	Feed	CH_4	0.509	Kg Kg^{-1}	Naphtha Cracking* [6]	7.10E-01	3.61E-01
	Feed	Sulfur	0.984	Kg Kg^{-1}	ecoinvent 3.71	1.44E-01	1.42E-01
	Feed	TFA	0.014	Kg Kg^{-1}	ecoinvent 3.71	cut-off	-
	Utility	Process Water	0.560	Kg Kg^{-1}	ecoinvent 3.71	1.88E-04	1.05E-04
	Utility	Electricity	1.394	kWh Kg^{-1}	ecoinvent 3.71	6.13E-01	8.54E-01
	Utility	Steam (LP)	15.677	MJ Kg^{-1}	ecoinvent 3.71	5.42E-02	8.50E-01
	Utility	Steam (HP)	2.649	MJ Kg^{-1}	ecoinvent 3.71	5.42E-02	1.44E-01
	Utility	Cooling water (Power)	0.212	kWh Kg^{-1}	ecoinvent 3.71	6.13E-01	1.30E-01
	Utility	Cooling water (Evaporation)	11.298	Kg Kg^{-1}	ecoinvent 3.71	6.70E-05	7.57E-04
Output	Waste	Purge gas	0.041	Kg Kg^{-1}	Designated waste incineration**	3.43E-01	1.40E-02
	Waste	CO_2	0.005	Kg Kg^{-1}	Direct emission	1.00E+00	4.63E-03
	Waste	Waste slurry	0.001	Kg Kg^{-1}	Designated waste incineration**	3.43E-01	1.75E-04
Product		Methanol	1.000	Kg Kg^{-1}			5.6E-01
		Sulfuric acid	3.046	Kg Kg^{-1}			5.7E-01

*Product carbon footprint is obtained from carbon allocation for naphtha carking plant. Allocation is performed by methane byproduct weight proportional to that of ethylene product.

** Data for carbon emission from the designated waste incineration is obtained from ez-EPD (Environmental Product Declaration) provided by Ministry of Environment, Korea, Site: http://eng.me.go.kr/eng/web/main.do)

Supplementary References

1 Lee, H. W. *et al.* Pt black catalyzed methane oxidation to methyl bisulfate in H2SO4-SO3. *Journal of Catalysis* **374**, 230-236 (2019).

2 Bradford, E., Schweidtmann, A. M. & Lapkin, A. Efficient multiobjective optimization employing Gaussian processes, spectral sampling and a genetic algorithm. *Journal of global optimization* **71**, 407-438 (2018).

3 Kim, K., Kim, J., Kim, C., Lee, Y. & Lee, W. B. Robust Design of Multicomponent Working Fluid for Organic Rankine Cycle. *Industrial Engineering Chemistry Research* **58**, 4154-4167 (2019).

4 Kim, K. *et al.* Data-driven pilot optimization for electrochemical CO mass production. *Journal of Materials Chemistry A* **8**, 16943-16950 (2020).

5 Biegler, L. T., Grossmann, I. E. & Westerberg, A. W. Systematic methods for chemical process design. (1997).

6 Haribal, V. P., Chen, Y., Neal, L. & Li, F. Intensification of ethylene production from naphtha via a redox oxy-cracking scheme: Process simulations and analysis. *Engineering* **4**, 714-721 (2018).
